# TIMP-2 regulates proliferation, invasion and STAT3-mediated cancer stem cell-dependent chemoresistance in ovarian cancer cells

**DOI:** 10.1186/s12885-020-07274-6

**Published:** 2020-10-06

**Authors:** Ruth M. Escalona, Maree Bilandzic, Patrick Western, Elif Kadife, George Kannourakis, Jock K. Findlay, Nuzhat Ahmed

**Affiliations:** 1 Department of Obstetrics and Gynaecology, University of Melbourne, Melbourne, VIC 3050 Australia; 2 Centre for Reproductive Health, Hudson Institute of Medical Research, and the Department of Molecular and Translational Science, Monash University, Melbourne, VIC 3168 Australia; 3 Fiona Elsey Cancer Research Institute, Ballarat, 3353 Australia; 4 Centre for Cancer Research, Hudson Institute of Medical Research, and the Department of Molecular and Translational Science, Monash University, Melbourne, VIC 3168 Australia; 5 Federation University Australia, Vic, Ballarat, 3010 Australia

**Keywords:** Ovarian cancer, MMPs, TIMP-2, Cancer stem cells, STAT3, Proliferation, Invasion, Chemosensitivity

## Abstract

**Background:**

The metzincin family of metalloproteinases and the tissue inhibitors of metalloproteinases (TIMPs) are essential proteins required for biological processes during cancer progression. This study aimed to determine the role of TIMP-2 in ovarian cancer progression and chemoresistance by reducing TIMP-2 expression in vitro in Fallopian tube secretory epithelial (FT282) and ovarian cancer (JHOS2 and OVCAR4) cell lines.

**Methods:**

FT282, JHOS2 and OVCAR4 cells were transiently transfected with either single or pooled TIMP-2 siRNAs. The expression of different genes after TIMP-2 knock down (T2-KD) or in response to chemotherapy was determined at the mRNA level by quantitative real time PCR (qRT-PCR) and at the protein level by immunofluorescence. Sensitivity of the cell lines in response to chemotherapy after TIMP-2 knock down was investigated by 3-(4,5-dimethylthiazol-2-yl)-2,5-diphenyltetrazolium bromide (MTT) and 5-Ethynyl-2′-deoxyuridine (EdU) assays. Cell invasion in response to TIMP-2 knockdown was determined by xCELLigence.

**Results:**

Sixty to 90 % knock down of TIMP-2 expression was confirmed in FT282, OVCAR4 and JHOS2 cell lines at the mRNA and protein levels. TIMP-2 knock down did not change the mRNA expression of TIMP-1 or TIMP-3. However, a significant downregulation of MMP-2 in T2-KD cells occurred at both the protein and activation levels, compared to Control (Cont; scrambled siRNA) and Parental cells (P, transfection reagent only). In contrast, membrane bound MT1-MMP protein levels were significantly upregulated in T2-KD compared to Cont and P cells. T2-KD cells exhibited enhanced proliferation and increased sensitivity to cisplatin and paclitaxel treatments. Enhanced invasion was observed in the T2-KD-JOSH2 and OVCAR4 cells but not in T2-KD-FT282 cells. Treatment with cisplatin or paclitaxel significantly elevated the expression of TIMP-2 in Cont cells but not in T2-KD cells, consistent with significantly elevated expression of chemoresistance and CSC markers and activation of STAT3. Furthermore, a potent inhibitor of STAT3 activation, Momelotinib, suppressed chemotherapy-induced activation of P-STAT3 in OVCAR4 cells with concomitant reductions in the expression of chemoresistance genes and CSC markers.

**Conclusions:**

The above results suggest that TIMP-2 may have a novel role in ovarian cancer proliferation, invasion and chemoresistance.

## Background

Worldwide, of the 250,000 women who are diagnosed annually with ovarian cancer, about 140,000 die within 5 years. Ovarian cancer patients have the lowest survival rate among gynaecological cancers [1]. Despite a high initial response to debulking surgery and first line chemotherapy consisting of taxane and platinum-based drugs, almost all patients relapse within a few months with a chemoresistance-associated disease, a trend that remains stagnant for the last three to four decades [1]. Even though different mechanisms of chemoresistance have been described [2, 3], none have had an individual impact in a clinical setting. Hence, a more detailed knowledge of the biological mechanisms leading to chemoresistance is essential to achieve a better treatment outcome for persistent recurring ovarian cancer patients.

Matrix metalloproteinases (MMPs) are members of the metzincin family that facilitate extracellular matrix (ECM) degradation and thereby promote tumour angiogenesis, invasion and metastasis [4–6]. Conversely, tissue inhibitors of MMPs (TIMPs) are multifunctional proteins, which belong to a family of secreted and ECM bound proteins that naturally inhibit the proteolytic activity of MMPs [7, 8]. The four TIMP family members, TIMP-1, − 2, − 3 and − 4 share a substantial homology in their sequences [8]. Of all the TIMPs, TIMP-2 only interacts with a cell-membrane bound MMP, commonly known as MT1-MMP and can act as either an initiator or an inhibitor of MMP-2 activation. As an initiator of MMP-2 activation, the catalytic domain of MT1-MMP binds to the N-terminal region of TIMP-2. This leaves the C-terminal region of TIMP-2 free for binding to the hemopexin-like domain of pro-MMP-2 [9]. This ternary MT1-MMP-TIMP2 complex facilitates assembly of the extracellular secreted pro-MMP-2 on the cell surface in close proximity to TIMP-free active MT1-MMP. TIMP-free MT1-MMP then cleaves the pro-peptide from cell membrane bound pro-MMP-2 to produce mature MMP-2. In cases where TIMP-2 acts as an inhibitor of MMP-2, the C-terminal end of TIMP-2 acts as a receptor for the C-terminal region of MMP-2, binding of which prevents the interaction of TIMP-2 with MT1-MMP, thereby preventing subsequent MMP-2 activation [10].

The expression of TIMP-2 is universal in most cell types where it functions as an endogenous inhibitor of MMPs [11]. In addition to its MMP-2 dependent functions, TIMP-2 can regulate signalling pathways by direct interaction with the cell surface receptors on normal and cancer cells [12, 13]. TIMP-2 also mediates anti-angiogenic effects by inhibiting endothelial cell migration and invasion through α3β1 integrin [14]. The tumour microenvironment may provide paracrine cues that regulate these TIMP-2-dependent roles in cancer cells [15].

The concept that cancer stem cells (CSCs) are small populations of slowly proliferating, drug resistant cells that contribute to tumour initiation and progression, relapse and metastasis has gained substantial importance [16, 17]. Standard cancer therapies usually target and kill actively proliferating cancer cells thereby reducing tumour burden and decreasing cancer-associated symptoms. This however, does not remove the small population of drug-resistant CSCs. As a result, CSCs can contribute to persistent residual disease even after effective anti-cancer treatments. Hence, it has been proposed that CSCs initiate cancer relapse, which ultimately results in mortality. We and others, have previously shown a correlation between activated STAT3 pathway and the existence of chemoresistance-associated CSCs in residual tumours [18, 19, 20, 21]. Activated STAT3 was shown to be essential for the sustenance of glioblastoma stem cells [22], fast proliferating intestinal [23] and mammary stem cells [24]. In addition, the activated STAT3 pathway has been associated with the progression and chemoresistance in ovarian and other cancers [25, 26].

In this study, we report significantly elevated expression of TIMP-2 in high-grade serous compared to benign ovarian tumours. By using siRNA, we examined the effect of TIMP-2 knock down (T2-KD) on MT1-MMP and MMP-2 expression levels, proliferation, invasion and chemosensitivity in Fallopian tube secretory epithelial cells and two ovarian cancer cell lines. We demonstrate that T2-KD cells are associated with increased expression of MT1-MMP and decreased expression and activity of MMP-2 in ovarian cancer and Fallopian tube epithelial cells. The knock down of TIMP-2 resulted in enhanced cell proliferation and increased sensitivity to chemotherapy treatments in ovarian cancer and Fallopian tube epithelial cells. However, increased invasion was only observed in ovarian cancer T2-KD cells but not in T2-KD Fallopian tube cells. Increased sensitivity to chemotherapy in T2-KD cancer cells was associated with an inability to activate the STAT3 pathway and a consequent deficit in chemotherapy resistance and CSC traits. These data reveal a potential role of TIMP-2 in the regulation of proliferation, invasion and chemoresistance in ovarian cancer cells and identifies TIMP-2 as a potential target to circumvent chemoresistance in ovarian cancer.

## Methods

### Patients and tissue samples

Ovarian tumours were acquired from ovarian cancer patients admitted to The Royal Women’s Hospital after procuring written consents under officially accepted ethical approval (Ethics approval #09/09) by the Research and Ethics Committee of Royal Women’s Hospital, Melbourne, Australia. High-grade serous epithelial ovarian tumours were obtained from seventeen patients undergoing surgery at the Oncology Dysplasia Unit of The Royal Women’s Hospital, Melbourne, Australia. Eight normal/benign samples were obtained from patients undergoing abdominal hysterectomy or bilateral salpingo-oophorectomy due to pre-existing medical conditions. At the time of collection, tissues were fixed in 4% paraformaldehyde. Information on tumour grade, stage and histopathology for individual tumour was obtained from pathology reports. The clinical information on each tumour sample is described in Table 1. Patients recruited in this study did not undergo treatment with chemotherapy, immunotherapy or radiation.

**Table 1:** Patient data

	Pathologist diagnosis	Cancer type	Tumour type	FIGO stage	Silverberg classification	WHO classification	CA125 levels (from pathology reports)	Peripheral blood CA125 at diagnosis	Ascites present at diagnosis	Genetic information	Survived from diagnosed till death
1	Benign sclerosis tumour	Benign	Benign	--	--	--	Increased	69	no	Nil Ca	--
2	Normal ovaries and Fallopian tubes	Normal	Normal	--	--	--	N/A	N/A	no	BRCA1+	--
3	Benign serous cystadenoma	Normal- Peutz-Jegher's syndrome	Benign	--	--	--	N/A	N/A	no	Peutz-Jegher's syndrome	--
4	Fibroma-mitotically active	Benign	Benign	--	--	--	N/A	>7	no	Nil Ca	--
5	Serous cystadenofibroma	Large multi cystic ovarian mass (benign)	Benign	--	--	--	N/A	N/A	no	Nil Ca	--
6	Serous cystadenofibroma	Cyst benign	Benign	--	--	--	N/A	6	no	Diagnosed with Breast Ca	--
7	Serous cystadenofibroma	Large ovarian cyst (benign)	Benign	--	--	--	N/A	>27	no	No info. available	--
8	Simple serous cyst	Benign	Benign	--	--	--	N/A	N/A	no	HNPCC carrier (colorectal Ca)	--
1	Serous carcinoma	Papillary serous cystadenocarcinoma	Malignant	IIc	G3	II	300	223	no	No (mother kidney Ca, Breast Ca)	--
2	Serous carcinoma	Papillary serous cystadenocarcinoma	Malignant	IIc	G3	II	N/A	24	yes	No (sister died melanoma)	--
3	Serous carcinoma	Serous cystadenocarcinoma NOS	Malignant	IIc	G3	II	109	107	no	Nil Ca	7 years 7 months
4	Serous carcinoma	Papillary serous cystadenocarcinoma	Malignant	IIc	G3	II	N/A	N/A	unknown	Nil Ca	--
5	Serous carcinoma	Serous cystadenocarcinoma NOS	Malignant	IIb	G2	II	1404	1404	no	Nil Ca	--
6	Serous carcinoma	Serous cystadenocarcinoma NOS	Malignant	IIIa	G3	II	N/A	104	yes	BRCA2+ve	5 years 1 months
7	Serous carcinoma	Papillary serous cystadenocarcinoma	Malignant	IIIc	G2	II	N/A	N/A	yes	Nil Ca	--
8	Serous carcinoma	Papillary serous cystadenocarcinoma	Malignant	IIIc	G2	II	1200	N/A	N/A	Nil Ca	--
9	Serous carcinoma	Papillary serous cystadenocarcinoma	Malignant	IIIc	G2	II	N/A	428	yes	N/A	3 years 7months
10	Serous carcinoma	Papillary serous cystadenocarcinoma	Malignant	IIIc	G3	II	N/A	4831	yes	Nil Ca	4 years 1months
11	Serous carcinoma	Papillary serous cystadenocarcinoma	Malignant	IIIc	G3	II	1230	1113	yes	BRCA2 +ve	--
12	Serous carcinoma	Carcinoma NOS	Malignant	IIIc	G3	II	N/A	3058	yes	BRCA1 +ve	2 years 7months
13	Serous carcinoma	Serous cystadenocarcinoma NOS	Malignant	IIIc	G3	II	Increased	3025	yes	Nil Ca	2 years 5months
14	Serous carcinoma	Serous cystadenocarcinoma NOS	Malignant	IIIc	G3	II	Increased	957	yes	Nil Ca	--
15	Serous carcinoma	Serous surface papillary carcinoma	Malignant	IIIc	G3	II	374	397	yes	BRCA2 +ve	4 years 7months
16	Serous carcinoma	Papillary serous cystadenocarcinoma	Malignant	IV	G2	II	N/A	3187	yes	sister with Ca	6 years 10months
17	Serous carcinoma	Serous cystadenocarcinoma NOS	Malignant	IV	G2	II	Raised	333	N/A	BRCA2 carrier	3years 11months

*II* Type II classification/ High grade tumour, *Nil Ca* negative for BRCA mutations and no family history of Cancer, *Ca* Cancer, *N/A* no data available

### Immunohistochemistry and quantitative analysis of protein expression

Immunohistochemistry staining of tumours was outsourced to the Anatomical Pathology Laboratory Services at The Royal Children’s Hospital, Melbourne, Australia. Briefly, paraffin embedded tissue samples were sectioned at 4 μm thickness and stained using 1:100 TIMP-2 polyclonal antibody (PAB11827, Abnova, Taipei, Taiwan) and OptiView DAB IHC Detection kit (Ventana Medical Systems, Inc., Arizona, USA). The samples were processed on Ventana Benchmark Immunostainer (Ventana Medical Systems, Inc., Arizona, USA) as described previously [19]. Negative controls used in this study were prepared by incubating samples in diluent without primary antibodies followed by the secondary antibody. Sections of human placental and tonsil tissues were used in each slide as positive controls to determine the staining efficiency of the antibodies used. Stained slides were then scanned at X40 magnification by the Southern Health Tissue Bank at Monash Medical Centre (Victoria, Australia) using the Aperio Scanscope XT (Aperio-Leica Microsystems Pty Ltd) and imaged using the Aperio ImageScope v12.3.2.8013 software (Leica Biosystems Pathology Imaging 2003–2016). Sections were evaluated microscopically for positive DAB staining in conjunction with positive CA125 (Ventana Medical Systems, Inc., Arizona, USA) staining. Three to eight random areas were selected and DAB positivity over each of these areas was calculated and divided by the average of negative control of each group. Results were plotted on a bar graph using PRISM software.

### Cell culture

Two established ovarian cancer cell lines were used for this study. JHOS2 (cell line derived from a primary tumour of a patient with high-grade serous cystadenocarcinoma, original repository: RIKEN, catalogue RCB 1521) [27, 28] and OVCAR4 (a cell line derived from the ascites of a patient diagnosed with ovarian serous adenocarcinoma, pre-treated with cyclophosphamide cisplatin and doxorubicin chemotherapies, Cellosaurus cell line, CVCL_1627) [29]. These cell lines were obtained from Professor David Bowtell (Peter MacCallum Cancer Centre, Parkville, Australia). The immortalised Fallopian tube secretory epithelial cell line, FT282, used as a non-cancer control, was a gift from Professor Ronny Drapkin (University of Pennsylvania) [30] and was obtained from Professor David Bowtell’s laboratory in Peter MacCallum Cancer Centre, Melbourne Australia.

OVCAR4 cells were maintained in RPMI-1640 (Sigma-Aldrich, Sydney, Australia); JHOS2 and FT282 were maintained in F-12 and DMEM medium (1:1). Each cell line medium was supplemented with L-glutamine (2 mM), and antibiotics (Fungizone, streptomycin and penicillin 1% v/v) and FBS (10% v/v) with the exception of the FT282 cell line which was supplemented with Ultroser™ G serum substitute (PALL, Life Sciences, NY, USA) instead of FBS. JHOS2 culture medium was supplemented with non-essential amino acids (1% v/v). Cell lines were maintained at 37 °C in 5% CO_2_. All cell lines were passaged at least twice a week once they reached a confluence of 65–80%.

### Transient transfections of cell lines

Three unique 27mer small interfering RNA (siRNA A, B, C) duplexes directed against human TIMP-2 (OriGene Technologies, SR304838, MD, USA) and a pooled siRNA (A + B + C) directed against TIMP-2 were used to knock down TIMP-2 expression (T2-KD) in FT282, JOSH-2 and OVCAR4 cell lines. A Universal non-targeting siRNA duplex was used as a Control (Cont) (OriGene Technologies, SR30004, MD, USA) in these experiments. To avoid off-target effects, the lowest TIMP-2 siRNA concentrations were optimized for each cell line (range tested was from 1 nM to 10 nM) and transfected cells were collected for RNA analysis 48 h after transfection. Transfection efficiency for each cell line was evaluated by using 15 nM siGLO™ Red Transfection Indicator (Dharmacon) as per manufacturer’s instructions. Parental cells (P) were cells treated with transfection reagent but no siRNA. Untreated cells (Unt) are parental cells without any treatment.

### Immunofluorescence

Immunofluorescence analysis was conducted on cell lines as described previously [31]. Briefly, 1 X 10^4^ cells were cultured overnight on 8-well chamber slides (Lab-Tek II Chamber Slide System) in complete growth medium at 37 °C in 5% CO_2_. Next day the cells were fixed with paraformaldehyde (PFA)/PBS solution, permeabilized using 0.1 (v/v) Triton X-100 (Sigma-Aldrich) in PBS, washed with cold PBS and incubated for 2 h with blocking buffer (1% BSA/PBS) followed by primary antibody treatment overnight at 4 °C (Table 2). Cells were stained with appropriate secondary antibodies (1:200 dilutions) (Table 2) in blocking buffer for 2 h. DAPI (4′,6-diamidino-2-phenylindole) (Invitrogen, Carlsbad, USA) was used to stain cellular nuclei at a 1:2000 dilution for 10 min at room temperature. Fluorescence imaging was visualized using an OLYMPUS BX53F upright microscope (Olympus, Tokyo, Japan) and images were taken using an Olympus DP70 camera and the Olympus CellsSens Dimension version 1.7.1 software (Olympics Corporation). The microscope filter parameters used for the individual fluorophore are provided in the supplementary Table 1. To avoid biased measurements, equal intensity acquisition parameters were set prior to imaging (for example the fluorophores DAPI acquisition time was set at 5 ms, Alexa 488 at 300 ms, Alexa 568 at 150 ms and Alexa 594 at 450 ms). The intensity units were measured using FIJI analysis software [ImageJ software 1.51j8 (Wayne Rasband National Institute of Health, USA)] according to DAPI location. This was repeated at least 4–9 times for each photograph and at least two images were taken for each well.

**Table 2:** Antibodies used in immunofluorescence study

Antibody	Host species	Catalogue number and company	Dilution used
ERCC1	Mouse	Ab2356, Abcam	1:98
Anti-betaIII	Rabbit	Ab18207, Abcam	1:180
Anti-TIMP2 antibody [3A4]	Mouse	Ab1828, Abcam	1:20
MT1-MMP	Rabbit	13130S, Cell signaling	1:200
OCT4	Rabbit	Ab19857, Abcam	1:180
EpCAM (VU109)	Mouse	2929, Cell signaling	1:800
STAT3 (124H6) (total STAT3)	Mouse	9139S, Abcam	1:1600
Phospho-Stat3 (Tyr705)	Mouse	9131S, Cell signaling	1:200
MMP2	Goat	AF902, R&D systems	1:40
CD44 (T2-F4)	Rat	Ab40983, Abcam	1:100
CD133/Prom1	Rabbit	Ab19898, Abcam	1:200
Alexa Fluor™ 488 Goat Anti-Rabbit IgG (H+L)	Goat	A32731, Life Technology	1:200
Alexa Fluor™ 568 Donkey Anti-Mouse IgG (H+L)	Donkey	A10037, Life Technology	1:200
Alexa Fluor™ 594 Donkey Anti-Goat IgG (H+L)	Donkey	A32758, Life Technology	1:200
Alexa Fluor™ 488 Goat Anti-Rat IgG (H+L)	Goat	A-11006, Life Technology	1:200

**Table 3:** Primer sequences, accession numbers, fluorescence capture of the genes analysed

Name	Primer sequences (5' - 3')	Accession number	Fluorescence capture (°C)
18S rRNA	F GTAACCCGTTGAACCCCATT R CCATCCAATCGGTAGTAGCG	NR_003286.1	78
TIMP-1	F TGACATCCGGTTCGTCTACA R GTTTGCAGGGGATGGATAAA	NM_003254.2	85
TIMP-2	F CCGCAACAGGCGTTTTGCAA R TCACTTCTCTTGATGCAGGC	NM_003255.4	85
TIMP-3	F TTCTGCAACTCCGACATCGT R ATGCAGGCGTAGTGTTTGGA	NM_000362.4	83
ERCC1	F TTGTCCAGGTGGATGTGAAA R GCTGGTTTCTGCTCATAGGC	NM_202001.2	83
TUBB3	F GGCCTTTGGACATCTCTTCA R ATACTCCTCACGCACCTTGC	NM_006086.3	88
CD44	F CCAATGCCTTTGATGGACCA R TGTGAGTGTCCATCTGATTC	NM_000610.3	81
OCT4A	F CTCCTGGAGGGCCAGGAATC R CCACATCGGCCTGTGTATAT	NM_002701.4	88
PROM1/ CD133	F ATTGGCATCTTCTATGGTTT R GCCTTGTCCTTGGTAGTGT	NM_006017	78
EpCAM	F CGTCAATGCCAGTGTACTTCAGTTG R TCCAGTAGGTTCTCACTCGCTCAG	NM_002354.2	80
MMP14/ MT1-MMP	F GCTCCGAGGGGAGATGTTTG R CAGCTCCTTAATGTGCTTGGG	NM_002428	83
MMP-2	F TTGACGGTAAGGACGGACTC R ACTTGCAGTACTCCCCATCG	NM_004530.4	81
E-CAD/ CDH1	F GGCACAGATGGTGTGATTACAG R GTCCCAGGCGTAGACCAAGAAA	NM_004360.3	75
N-CAD/ CDH2	F AAACAGCAACGACGGGTTAG R CTTAGGATTGGGGGCAAAAT	NM_001792.3	78
VIM	F CCTACAGGAAGCTGCTGGAA R GGTCATCGTGATGCTGAGAA	NM_003380.3	75
COL12A1	F ACCTGTCACTGTTCGGGAAG R TGAGGGAAGTGCTGGTCTCT	NM_080645.2	80

### Gelatin Zymography

For zymography analysis, complete growth media was discarded and replaced by OPTIMEM media (GIBCO Life Technology, NY, USA). The serum free medium was collected and concentrated using 10 kDa Amicon ultra-4 spin columns (Merck-Millipore, Billerica, MA, USA). Secreted proteins in the conditioned medium were separated using SDS-PAGE gel (10%) containing 1% gelatin as described previously [32]. The gels were stained with Coomassie Blue, and distained at room temperature. To measure MMP activities, gels were incubated in 10 mM EDTA (Fisher, Fair Lawn, NJ USA) and a proteolytic inhibitor, as described previously [33]. Bands were visualized and analysed using the Fuji Film LAS-3000 gel doc system and the Image Lab software version 6.0.0 (Bio-Rad Laboratories, Inc., Gladesville, NSW, Australia). Semi-quantitative densitometry analysis was performed using Image J software 1.51j8 (Wayne Rasband National Institute of Health, USA).

### Proliferation assays

#### MTT assay

This was performed as described previously [19, 31]. Cells (3X10^4^) were seeded and transfected in 96-well plates on the same day and after 24 h, cell culture medium was replaced. Chemotherapy (paclitaxel or cisplatin) at different concentrations (0 to 320 μg/ml) was added to cells for 48 h after which culture medium was replaced and 100 μL of 3-(4,5-dimethylthiazol-2-yl)-2,5-diphenyltetrazolium bromide) (MTT) solution (Sigma-Aldrich) dissolved in 1x PBS solution (0.5 mg/ml, final concentration) (Sigma-Aldrich) was added to the cells. After 2 h incubation, cell culture medium was replaced with 100 μl of dimethyl sulfoxide (DMSO). Absorbance was read at OD595nm using the CLARIOstar Plate Reader (BMG Labtech, Germany) and MARS Data Analysis Computer Software (BMG Labtech, Mornington, Victoria, Australia).

#### EdU assay

This assay was performed using the Click-IT™ Plus EdU Flow Cytometry Assay Kit (Invitrogen/Thermo Fisher Scientific North Ryde, NSW, Australia) essentially as described previously [34]. Briefly, 2X10^4^ cells were grown and transfected in 24-well plates for 48 h prior to the assay. 5-Ethynyl-2′-deoxyuridine (EdU) was added at a final concentration of 10 μM and the cells were incubated for 1 h at 37 °C. Cells were trypsinized, centrifuged at 1200 rpm for 5 min and washed with 1% BSA in PBS and fixed in Click-IT fixative. Cell pellets were washed with Perm-wash buffer, stained with Alexa Fluor 647 Picoyl Azide, washed in Perm-wash and resuspended in Perm-wash containing 20 μg/ml propidium iodide. The cells were then analysed by flow cytometer using 633/635 nm excitation with a red emission filter for the detection of Alexa Fluor 647 Azide. Single cells were gated using forward scatter height vs area. Single cells were then plotted according to EdU/Alexa 647 Azide (Log scale, y-axis) to identify cells in S-phase based on incorporation of EdU and PI staining (linear scale, x-axis) and to separate cells in G0/G1, S-phase and G2/M based on DNA content. Cells for which EdU or Alexa 647 Azide were omitted were used as negative controls for EdU staining.

### Invasion assay

Cell invasion assay was performed by using the Roche xCELLigence DP instrument as described previously [35]. The upper chamber of the 16-well CIM plate (Roche, NSW, Australia) was coated with 20 μl of matrigel and left to set for 30 min at 37 °C. Cells (4X10^4^) suspended in 130ul Gibco® Opti-MEM™ Media (Thermo-Fisher Scientific, NSW, Australia) were seeded on the top compartment of the pre-equilibrated 16-well CIM plate (Roche). Readings were taken every 15 min for ~ 40 h. Each plate contained two duplicate wells and each experiment was repeated 3 times. Mean values from three experiments on each Cont and T2-KD cell lines are illustrated graphically using PRISM software. Linear regression analysis of two slopes arising from Cont and T2-KD cells were used to obtain significant values.

### RNA extraction, quantitative and relative real-time PCR (qRT-PCR)

RNA was extracted from chemotherapy-treated (cisplatin or paclitaxel) and untreated cell lines using TRIzol® reagent (Ambion-Life Technologies, Carlsbad, CA, USA) followed by the chloroform: phenol method as described previously [18]. Five hundred ng of total RNA was reverse transcribed using the high capacity cDNA Reverse Transcription Kit (Applied Biosystems, CA, USA) and qRT-PCR amplification was performed using the Applied Biosystems ViiA 7 Real-Time PCR (Thermo Fisher Scientific, NSW, Australia) as described previously [18]. Table 3 lists the sequences and accession numbers of genes analysed. Data are presented as absolute values (fg) normalized to 18S (Fig. 2a) [36] or relative expression normalized to housekeeping gene 18S [Fig. 3 (b-c), Fig. 5 (a-d), Fig. 6 (c-e), Fig. 7 (b-c), Fig. 8 (a-b) and Fig. 9 (b-e)].

### Statistical analysis

When only two treatment groups were compared, an unpaired Man-Whitney’s non-parametric t-test was used. However, when more than two treatment groups were compared a One-Way ANOVA was used. Data are presented as mean ± standard error of the mean (SEM). xCELLigence data was analysed by linear regression analysis, and presented as the standard deviation (SD) of the mean. For statistical significance, the probability levels adopted were *p* < 0.05(*), *p* < 0.01(**), *p* < 0.001 (***) and *p* < 0.0001 (****). All data were analysed by Graph Pad PRISM software and Microsoft Excel 2016. All experiments were performed for a minimum of three times (unless otherwise indicated) in triplicate.

## Results

### The expression of TIMP-2 is significantly higher in human serous high-grade ovarian tumours compared to benign tumours

**Fig. 1:**
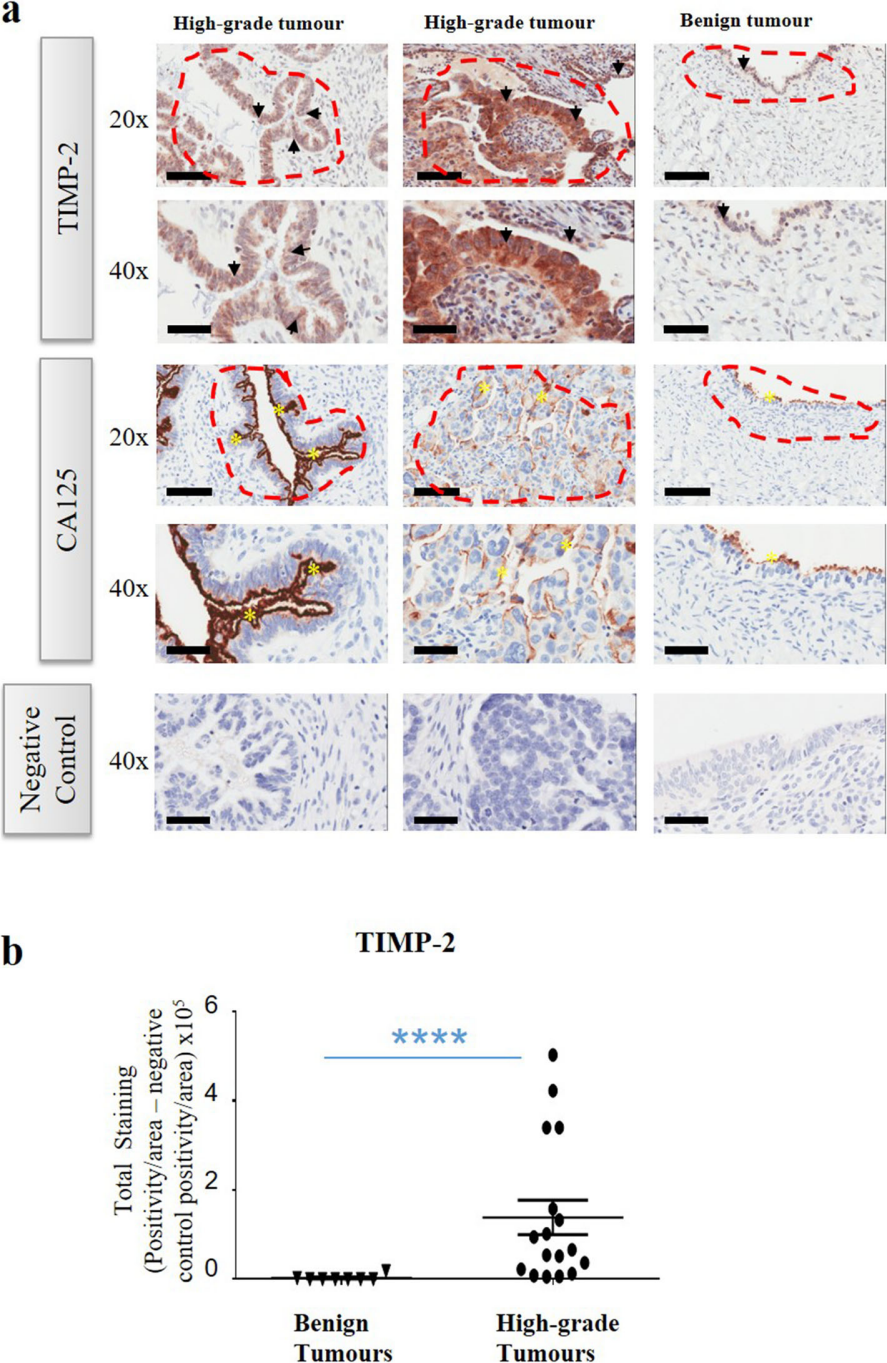
Expression and localization of TIMP-2 in high-grade serous ovarian tumours compared to benign serous tumours. **a** Representative images of TIMP-2 staining in primary ovarian serous high-grade, benign serous tumours and their matching controls. Colum 1 is a representative image of high-grade serous patient sample 9 (Table 1) and its matching control, column 2 is high-grade serous patient sample 17 (Table 1) and its matching control and the last column is a benign tumour from patient sample 5 (Table 1) and its matching control. Black arrowheads indicate positive TIMP-2 stained epithelial cells. Yellow asterisks indicate positive CA125 stained cells. Areas/regions included in the analysis were selected using CA125 marker to identify tumour positive areas, red dash lines indicated how areas were selected for analysis. Images are representative of high-grade (*n* = 17) and benign (*n* = 8) tumours. Magnification (20X), scale bar = 100um; Magnification (40X), scale bar = 50uM. **b** TIMP-2 expression was significantly upregulated in serous high-grade compared to benign serous tumours. Graph is a representative of DAB positivity over area divided by negative control for each patient tissue block described in Methods. Error bars are presented as mean ± of SEM. Significance is indicated by **** *p* < 0.0001, unpaired Man-Whitney’s non-parametric t-test

Twenty-five paraffin embedded tissues (Table 1), consisting of eight benign serous ovarian tumours and seventeen poorly differentiated high-grade serous tumours were analysed by immunohistochemistry using an anti-human TIMP-2 specific antibody. In > 90% tumours TIMP-2 expression was noted in the epithelial tumour cells also expressing CA-125, while in a few others stromal expression of TIMP-2 was also noted (Fig. 1a). Staining was mostly confined to the cytoplasm but in some cases, discrete membrane staining of the tumour cells was also evident (Fig. 1a). Diffuse TIMP-2 staining was noted in the stroma of some tumours, which was mainly cytoplasmic. In the case of benign tumours, only the ovarian surface epithelium stained positive for TIMP-2. The staining was weak and confined mainly to the cytoplasm. Expression of TIMP-2 was significantly enhanced in high-grade ovarian tumours compared to benign tumours (Fig. 1b).

### Expression of TIMPs, MMP-2 and MT1-MMP in a normal fallopian tube and two ovarian cancer cell lines

**Fig. 2:**
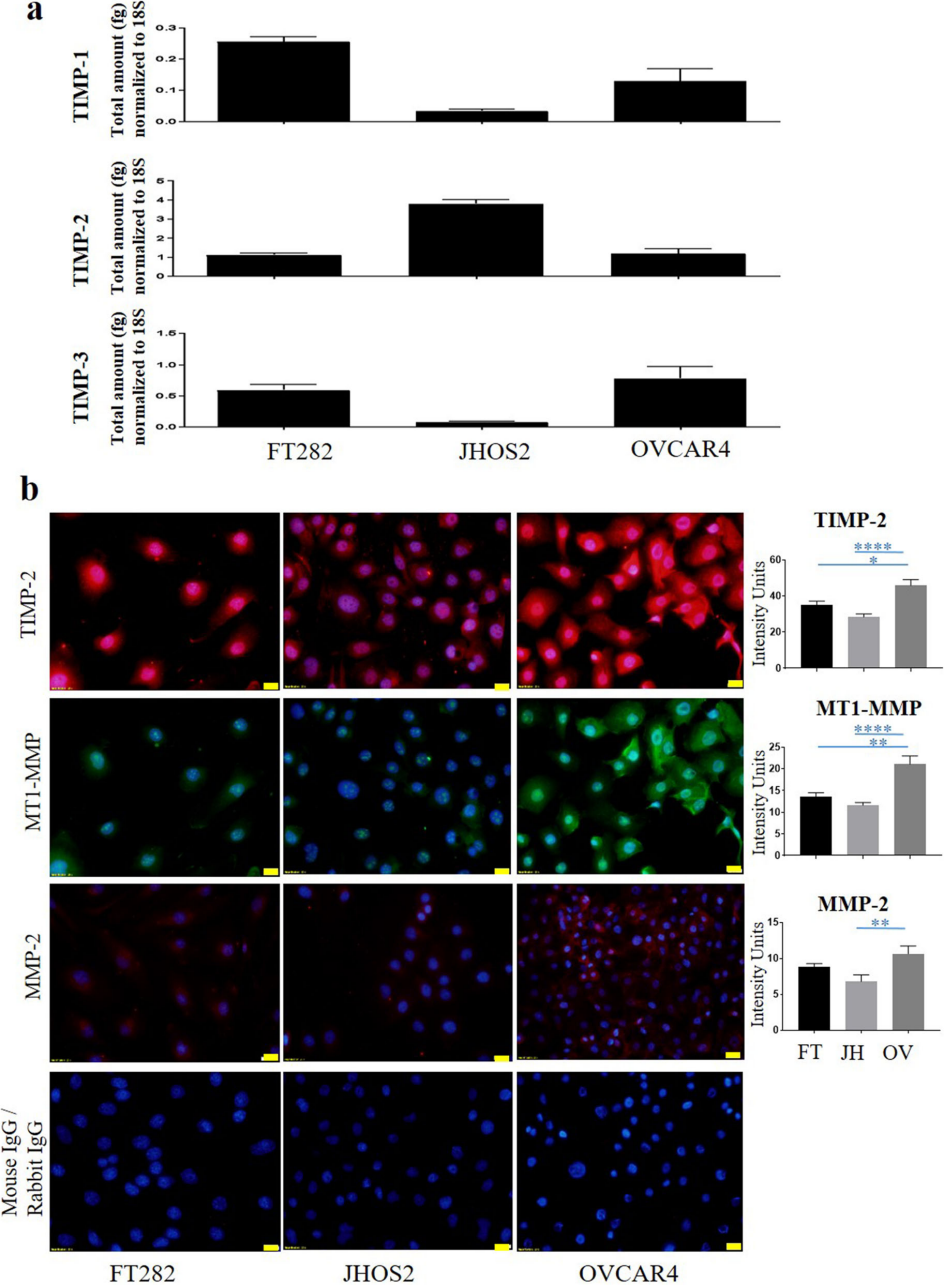
Expression of TIMP-1, TIMP-2, TIMP-3, MT1-MMP and MMP-2 in Fallopian tube secretory epithelial cell line and ovarian cancer cell lines. **a** The mRNA expression of TIMP-1, TIMP-2 and TIMP-3 in Fallopian tube secretory epithelial cell line, FT282, and ovarian cancer JHOS2 and OVCAR4 cell lines was evaluated by qRT-PCR. The experiment was repeated three times in triplicate. Graphs represent mean of total mRNA ± of SEM. **b** Protein expression of TIMP-2, MT1-MMP and MMP-2 was evaluated in FT282 (FT), and JHOS2 (JH) and OVCAR4 (OV) cell lines by immunofluorescence using rabbit polyclonal and mouse monoclonal antibodies as described in the Methods. Staining was visualized using the secondary anti-mouse Alexa 590 (red) for TIMP-2 and MMP-2 and anti-rabbit Alexa 488 (green) for MT1-MMP fluorescent-labelled antibodies and nuclei were detected by DAPI (blue) staining. The intensity of fluorescence was determined by using FIJI software. Images represent three independent experiments. 20X magnification; scale bar (in yellow) 20 μM. Significance between the groups was deduced by One-way ANOVA and is indicated by *p > 0.05, **p > 0.01, ****p < 0.0001

The mRNA expression of TIMP-1, − 2 and − 3 was measured in the ovarian cancer cell lines, JHOS2 and OVCAR4 and a Fallopian tube secretory epithelial cell line, FT282, which was used as a non-cancer control (Fig. 2a). All three cell lines expressed mRNA for TIMP-1, − 2 and − 3, with TIMP-2 mRNA expression being several-fold greater than the other TIMPs in each cell line. TIMP-2, MMP-2 and MT1-MMP protein was also detected using immunofluorescence in all three cell lines (Fig. 2b, Supplementary Fig. 1), but all three proteins were detected at higher levels in OVCAR4 cells compared to JHOS2 and FT282 cells.

### Reduction of TIMP-2 expression by siRNA had no significant effect on the expression of TIMP-1 and TIMP-3 mRNAs in the normal fallopian tube and ovarian cancer cell lines

**Fig. 3:**
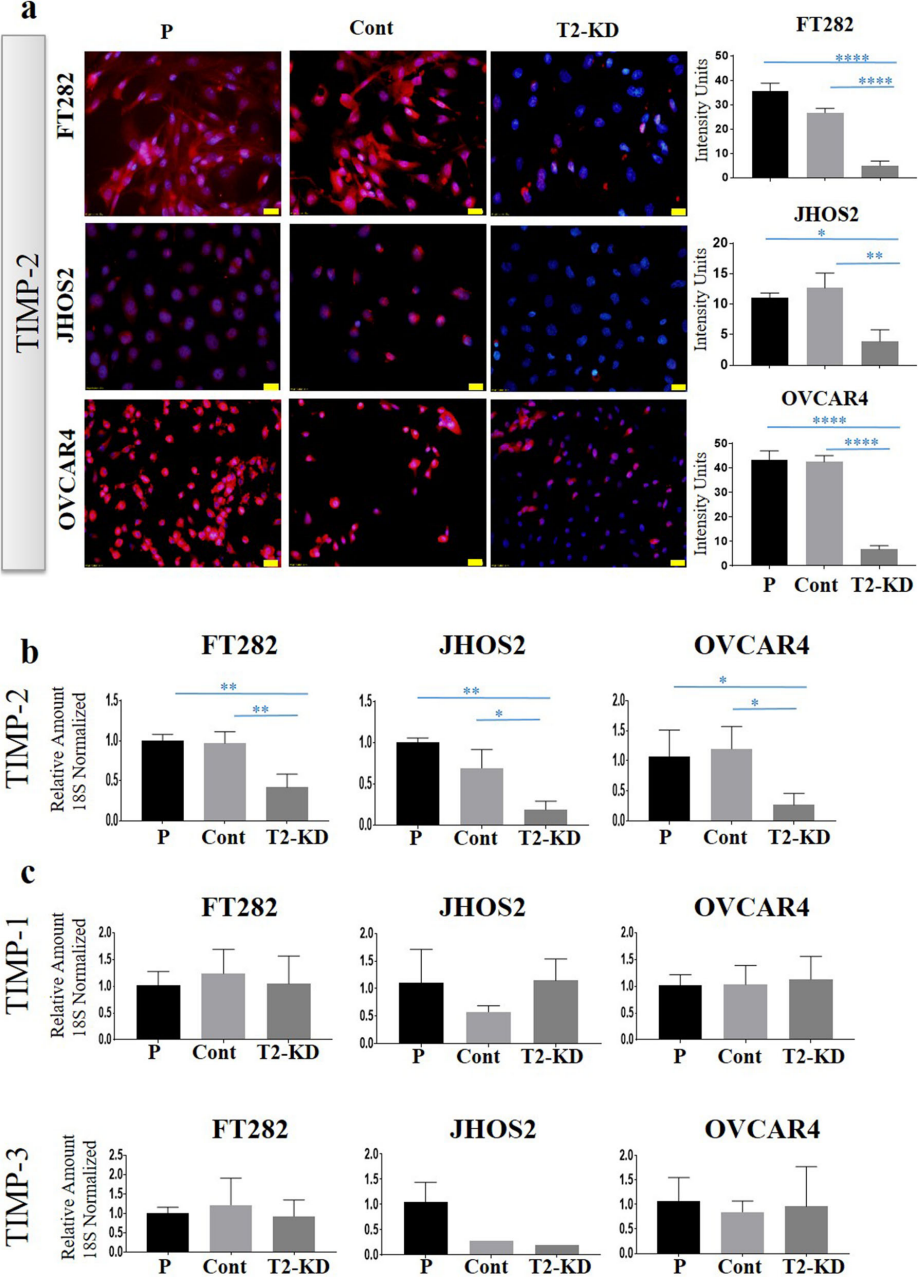
Reduction of TIMP-2 expression by siRNA: TIMP-2 expression knock down by siRNA transfection in FT282 (FT) and two ovarian cancer cell lines JHOS2 (JH) and OVCAR4 (OV) is described in the Methods. T2 is representative of a pool of all three TIMP-2 siRNAs at a 3 nM final concentration. **a** The expression of TIMP-2 at the protein level was deduced by immunofluorescence as described in Methods. Images are representation of merged DAPI (blue) and TIMP-2 (red) staining on individual cell line done in three passages in triplicate. The intensity of fluorescence was obtained using FIJI software. 20X magnification; scale bar (in yellow) 20 μM. P indicates parental cell line treated with transfection reagent, Cont are cells transfected with scrambled siRNA and T2-KD are TIMP-2 siRNA knock down cells. **b** mRNA expression of TIMP-2, **(c)** TIMP-1, and TIMP-3 was deduced after siRNA transfection in the representative cell lines by qRT-PCR as described in Methods. Graphs represents amount of mRNA relative to 18S + SEM derived from three experiments done in triplicate. Significance is determined by one-way ANOVA and indicated by *p < 0.05, **p < 0.01

To assess the function of TIMP-2 in cancer cell lines, the expression of TIMP-2 was knocked down in ovarian cancer cell lines JOSH2 and OVCAR4 and FT282 Fallopian cell line using three unique 27mer siRNA duplexes individually, or a pooled siRNA duplex directed against human TIMP-2 (T2-KD). A non-targeting universal siRNA was used as a Control (Cont) in these experiments. TIMP-2 protein expression was reduced by 90% in FT282 and 80% in both JOSH2 and OVCAR4 cell lines compared to Cont and Parental (P) cell lines using pooled TIMP-2 siRNA (Fig. 3a, Supplementary Fig. 2). Similar trends of TIMP-2 mRNA knockdown, 60% in FT282 cell line and 80% in both JOSH2 and OVCAR4 cell lines was observed using single 27mer TIMP-2 siRNA duplexes or with the pooled TIMP-2, siRNAs compared to Cont and Parental (P) cell lines (Fig. 3b, Supplementary Fig. 3A). However, knock down of TIMP-2 in FT282; JOSH2 and OVCAR4 cell lines had no significant effect on the mRNA expression of TIMP-1 and TIMP-3 (Fig. 3b, Supplementary Figs. 3 B-C).

### Reduction of TIMP-2 mRNA expression significantly enhanced the expression of MT1-MMP but decreased the activation and expression of MMP-2

**Fig. 4:**
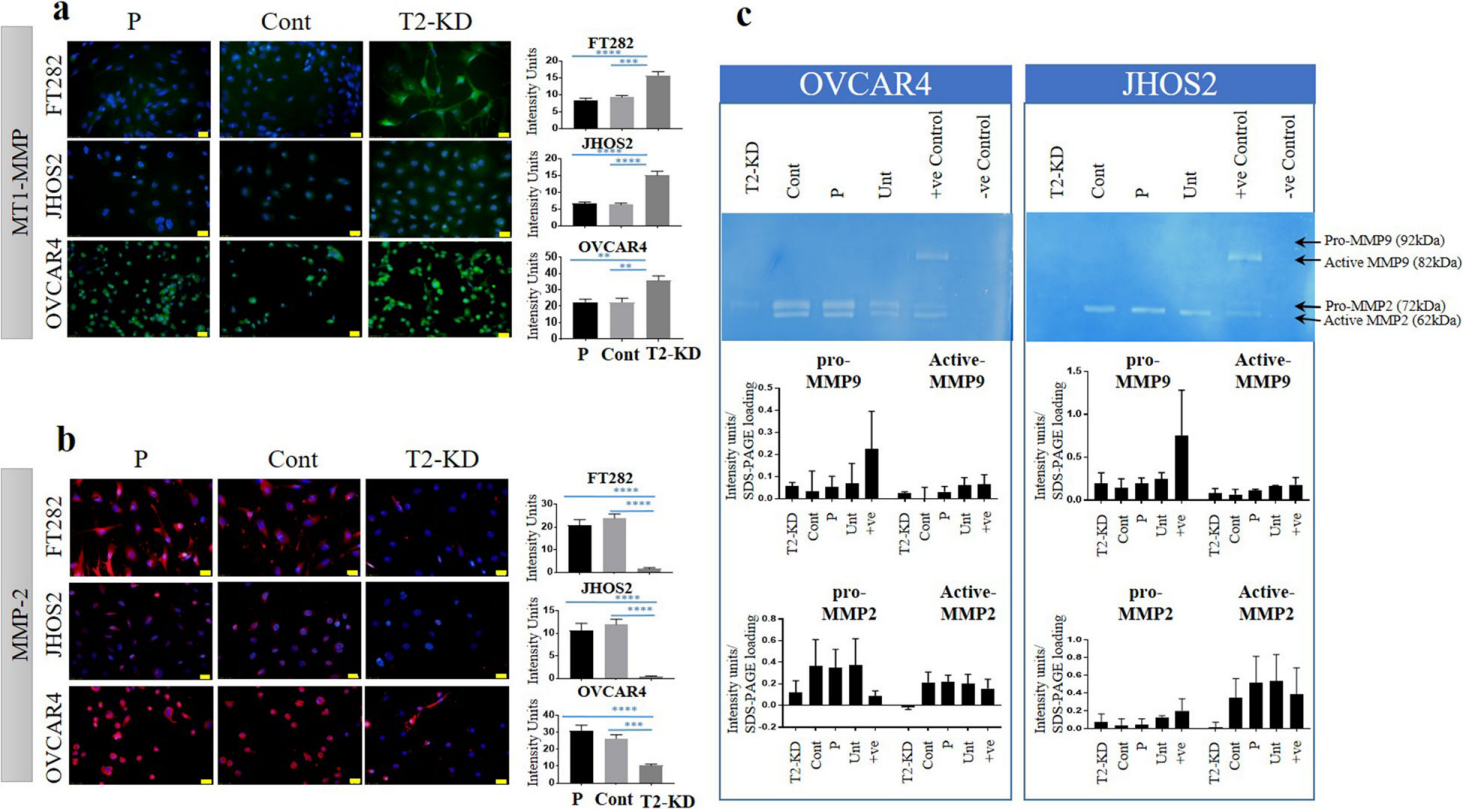
Expression of MT1-MMP, MMP-2 following reduction of TIMP-2 expression in cell lines. The protein expression of **a** MT1-MMP and **b** MMP-2 was evaluated by immunofluorescence in FT282, JHOS2 and OVCAR4 cell lines 48 h after siRNA knock down of TIMP-2 as described in Methods. T2-KD is representative of a pool of all three TIMP-2 siRNAs at a 3 nM final concentration. P are the parental cells treated with transfection reagent without siRNA treatment. Cont are parental cells transfected with scrambled siRNA. Images are representative of merged DAPI (blue) and MT1-MMP (green) or DAPI (blue) and MMP-2 (red) staining on individual cells performed on three passages in triplicates. The fluorescent intensity was obtained using FIJI software. Images represent three independent experiments, 20X magnification; scale bar (in yellow) 20 μM. Values are mean + SEM with significance deduced by using One-way ANOVA and indicated by **p < 0.01, ***p < 0.001; ****p < 0.0001). **c** MMP-2 activity in conditioned medium of OVCAR4 and JHOS2 cell lines was analysed by zymography as described in Methods. The treatment groups are T2-KD, Cont, P, Unt (untreated cells), +ve Control (serum conditioned media from HEY ovarian cancer cell line), −ve Control (serum reduced media, OPTIMEM). The image is representative of two experiments

As TIMP-2 is known to regulate MT1-MMP and MMP-2 by its direction interaction with these proteases, we next assessed the effect of TIMP-2 knockdown on the expression of MT1-MMP and MMP2. FT282, OVCAR4 and JHOS2 T2-KD cells had significantly enhanced expression of MT1-MMP compared to corresponding Cont and P cells as shown by immunofluorescence (Fig. 4a). However, MMP-2 expression was significantly downregulated after TIMP-2 knock down compared to Cont and P cells in the three cell lines studied (Fig. 4b). Consistent with the immunofluorescence analyses, zymography showed decreased activation of MMP-2 and lower expression of the pro-zymogen form of MMP-2 in T2-KD OVCAR4 and JHOS2 cells compared to Cont and P cells (Fig. 4c).

**Fig. 5:**
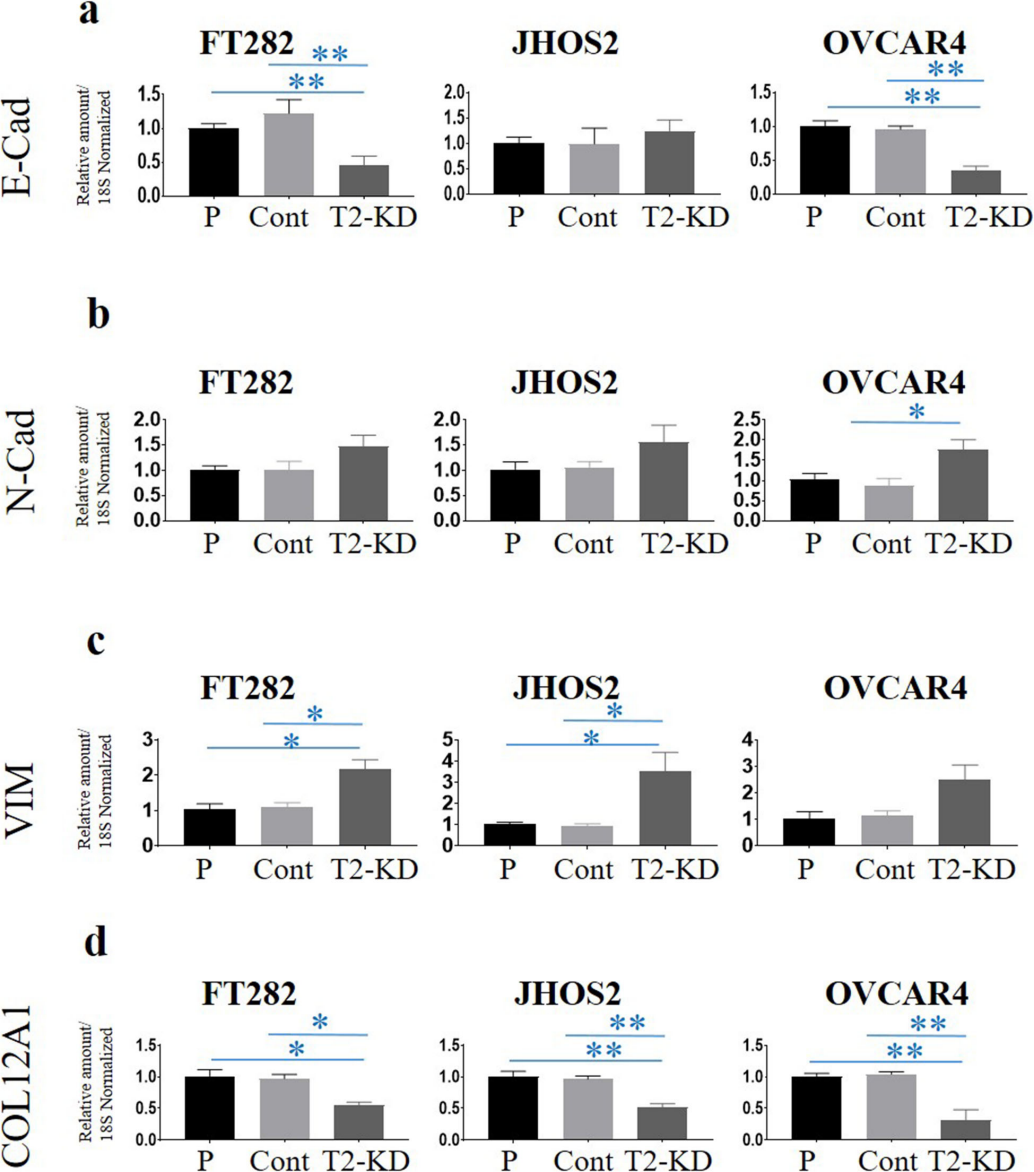
**a-d** mRNA expression of E-Cad, N-Cad, VIM and COL12A1 following reduction of TIMP-2 expression by siRNA. The expression of E-Cad, N-Cad, VIM and COL12A1 was deduced by qRT-PCR as described in the Methods. Graphs are representative of three experiments done in triplicate. Values are mean + SEM; significance was deduced by using One-way ANOVA and is indicated by *p < 0.05, **p < 0.01

As the expression of E-cadherin (E-Cad) has been shown to decrease with upregulation of MT1-MMP in many cancer cell lines [37], we compared the mRNA expression of E-Cad in T2-KD cells with the corresponding Cont and P cells (Fig. 5a). Significantly, decreased mRNA expression of E-Cad was observed in T2-KD FT282 and OVCAR4 cells compared to matched Cont and P cell (Fig. 5a). However, no such decrease in E-Cad expression was observed in T2-KD JHOS2 cells compared to its matched control. To determine if the decrease in E-Cad expression correlated with an increase in N-cadherin (N-Cad) and vimentin (VIM) expression, consistent with an epithelial mesenchymal transition (EMT) pattern in these cells, the mRNA expression of N-Cad and VIM was investigated. A significant increase in the expression of N-Cad was noted in T2-KD OVCAR4 cells and an increasing trend in T2-KD JOSH2 and FT282 cells, compared to Cont and P cells (Fig. 5b). The mRNA expression of VIM was significantly upregulated in JHOS-2 and FT282 T2-KD cells compared to their respective control cells (Fig. 5c). However, T2-KD OVCAR4 cells showed an increasing trend in VIM mRNA expression compared to corresponding controls (Fig. 5c).

In order to determine if the classical EMT pathway [38] was influenced by the knock down of TIMP-2, we evaluated the mRNA expression of three known EMT transcription factors, *SLUG*, *TWIST* and *SNAIL* in T2-KD FT282, JOSH2 and OVCAR4 cells, and their relevant Cont and P cells. However, there was no change in the expression of *SLUG* and *TWIST* in T2-KD FT282 and JOSH2 cells compared to Cont and P cells (Supplementary Fig. 4), and neither *TWIST* nor *SLUG* expression was detectable in OVCAR4 cell line.

As MT1-MMP dependent collagen degradation promotes remodelling of tissues during cancer progression [39], we determined the expression of collagen 12A1 (*COL12A1*, fibril associated collagen that modifies the interaction between collagen 1 and the surrounding matrix) in FT282, OVCAR4 and JHOS2 cells following TIMP-2 knockdown. The suppression of TIMP-2 in all three cell lines correlated with a significant decrease in *COL12A1* mRNA expression compared to their respective Cont and P cells (Fig. 5d). These results suggest that enhancement in MT1-MMP expression due to loss of TIMP-2 in FT282; OVCAR4 and JHOS2 cells may enhance collagen degradation-dependent extracellular matrix remodelling.

### Reduction of TIMP-2 expression significantly enhanced the proliferation of FT282 and JOSH2 and OVCAR4 cell lines but enhanced invasion was observed only in JOSH2 and OVCAR4 cell lines

**Fig. 6:**
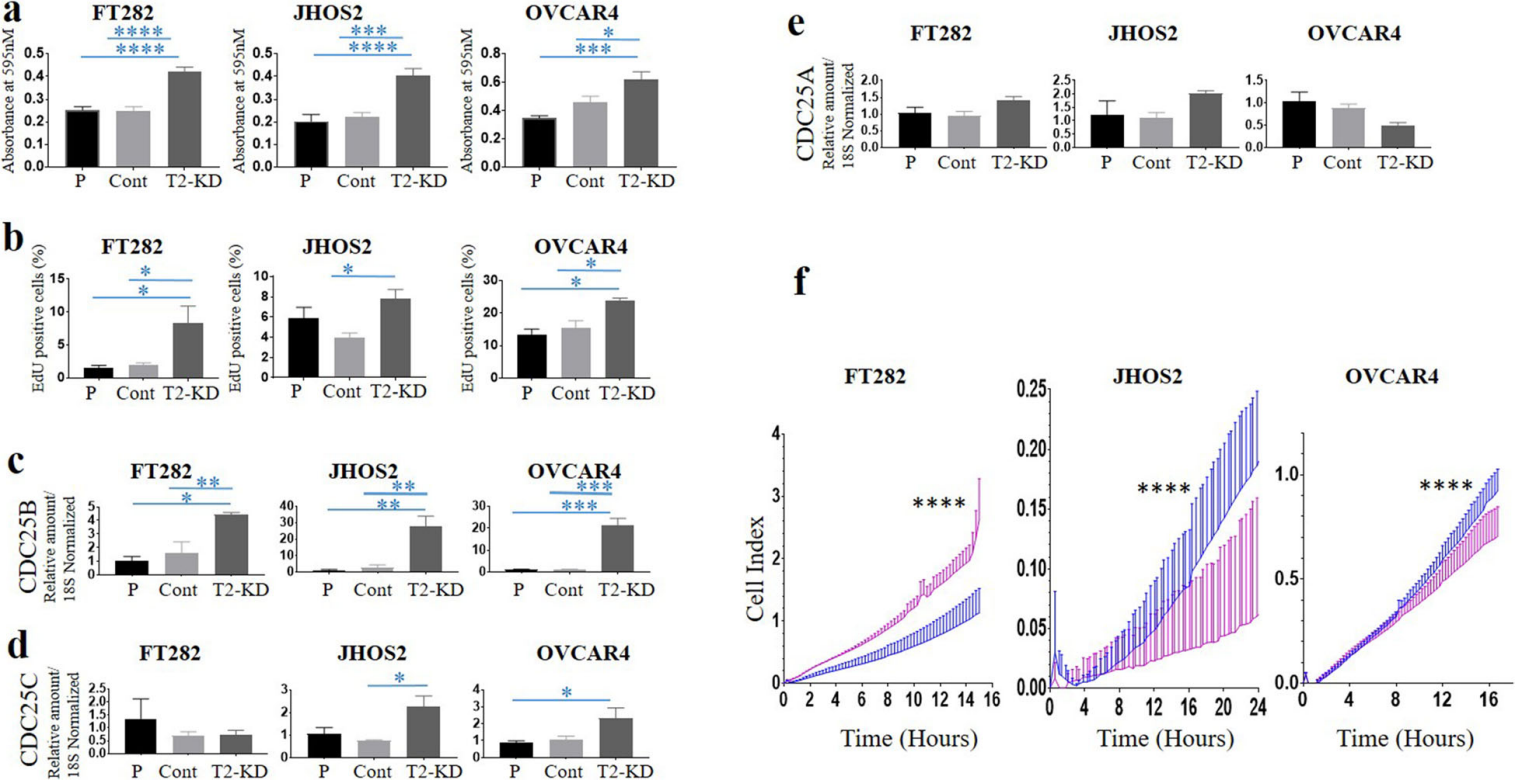
Effects of reduction of TIMP-2 mRNA on proliferation of FT282, JHOS2 and OVCAR4 cell lines. T2-KD is representative of a pool of all three TIMP-2 siRNAs. P are parental cells without siRNA treatment. Cont are parental cells transfected with scrambled siRNA. The experiments were performed 48 h after TIMP-2 knockdown by siRNA. **a** MTT assay was performed as described in Methods. Bar graphs represent mean ± SEM of 3 different MTT experiments. Significance was obtained using One-way ANOVA. *p < 0.05, ***p < 0.001, ****p < 0.0001. **b** Cells were stained for EdU and analysed as described in the Methods. Results are expressed as % of EdU positive cells (or cells in the S-phase of cell cycle progression). Bar graph represent *n* = 3 for each experiment. **c** mRNA expression of CDC25B; **d** CDC25C and **e** CDC25A were evaluated by qRT-PCR. Statistical significances were obtained using One-way ANOVA and are indicated by *p < 0.05, **p < 0.01, ***p < 0.001. **f** Effect of reduction in TIMP-2 mRNA on invasion of FT282, JHOS2 and OVCAR4 cell lines. T2-KD (blue line) is a representative of a pool of all three TIMP-2 siRNAs. Cont are parental cells transfected with scrambled siRNA (pink line). Invasion assays were assessed by xCELLigence real-time cell analysis. For assessment of invasion, the electrodes were coated with Matrigel and the bottom of the well contained reduced serum medium (OPTIMEM). Significance was assessed by linear regression analysis of two slopes; ****p < 0.0001

The proliferation of T2-KD FT282, OVCAR4 and JHOS2 and the relevant Cont and P cells was analysed 48 h after siRNA transfection using the MTT assay (Fig. 6a) and by measuring EdU (5-ethynyl-2′-deoxyuridine) incorporation into DNA using flow cytometry (Fig. 6b, Supplementary Fig. 5). Both assays revealed a significant enhancement in cellular proliferation in all T2-KD cells compared to the matched Cont and P cells. To further characterize the proliferative capacity of T2-KD cells, we evaluated the mRNA expression of the cell cycle enzymes, CDC25A, CDC25B and CDC25C [40]. The expression of CDC25B was significantly upregulated in T2-KD FT282, OVCAR4 and JHOS2 cells compared to their respective control cells (Fig. 6c). However, CDC25C was only upregulated in T2-KD OVCAR4 and JOSH2 cancer cells compared to their corresponding control cells (Fig. 6d) and there was no change in the expression of CDC25A in any of the T2-KD cells compared to its respective controls (Fig. 6e).

We next evaluated the invasive capacities of T2-KD cells by xCELLigence Real Time Cell Analysis. This revealed a significant enhancement of invasion through Matrigel by T2-KD JOSH2 and OVCAR4 cells compared to the respective control cells. In contrast, non-cancerous FT282 T2-KD cells showed significantly decreased invasion compared to the relevant control cells (Fig. 6f).

### Reduction of TIMP-2 expression enhanced cell sensitivity to chemotherapy

**Fig. 7:**
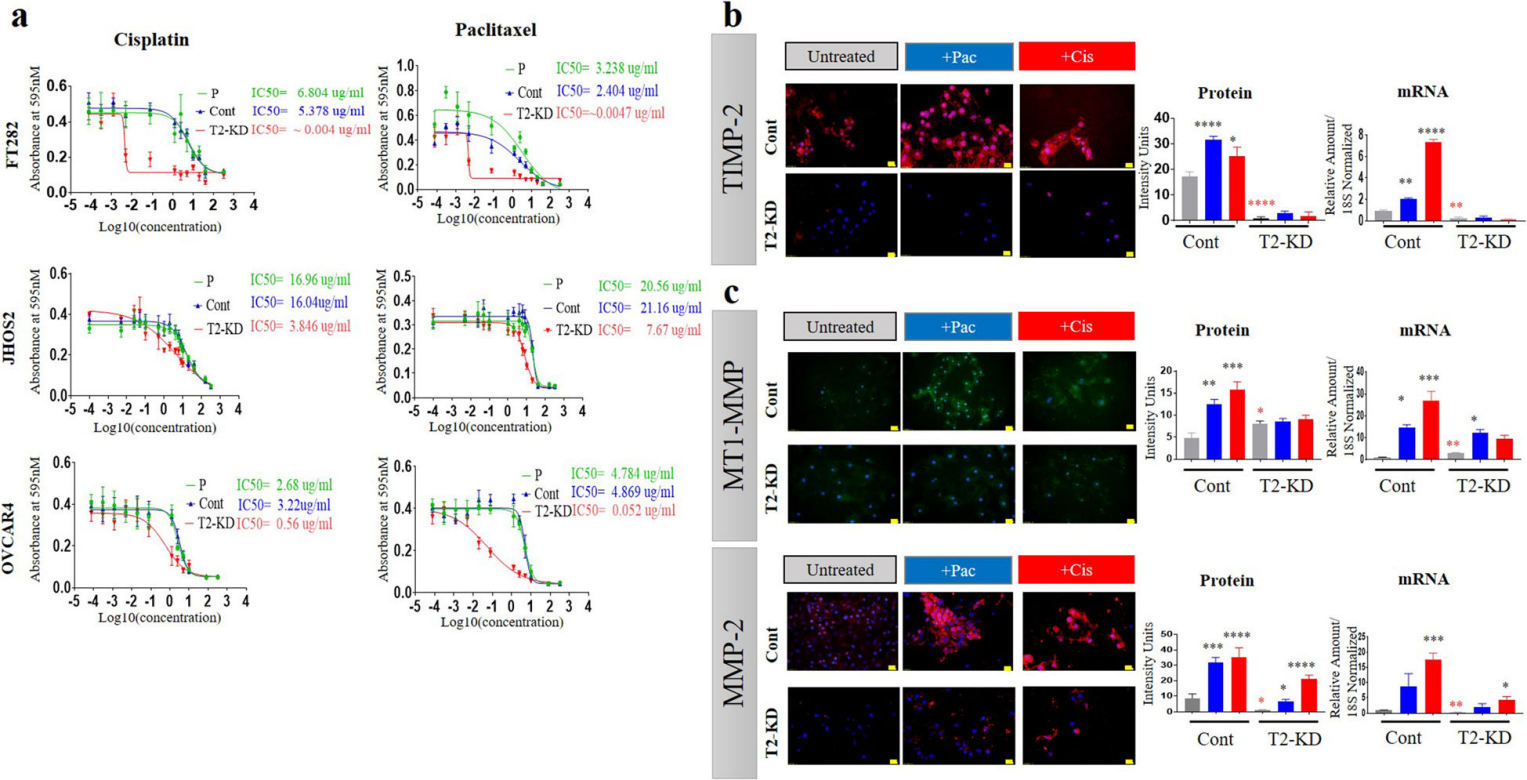
Effect of paclitaxel (pac) and cisplatin (cis) on the IC_50_ values and the expression of TIMP-2, MT1-MMP, MMP-2 in Cont and T2-KD cells. **a** Cell lines (FT282, JHOS2, and OVCAR4) were treated for 48 h with varying concentrations of either cisplatin or paclitaxel and their IC_50_ values (the concentration that kills 50% of the cells) was determined by MTT assay. T2-KD are TIMP-2 siRNA transfected cells (red line), P are parental cells without siRNA treatment (green line) and Cont are parental cells transfected with scrambled siRNA (blue line). Graphs are representative of three experiments done in triplicate. **b-c** The protein (by immunofluorescence) and mRNA (by qRT-PCR) expression of TIMP-2 (red), MTI-MMP (green) and MMP-2 (red) and co-stained DAPI (blue) in OVCAR4 Cont and T2-KD cells treated with their respective IC_50_ concentrations of cisplatin (red bars) and paclitaxel (blue bars). Images represent three independent experiments done in triplicate. For immunofluorescence images, magnification (20X); scale bar (in yellow) 20 μM. Significance was determined by one-way ANOVA *p > 0.05; **p > 0.01; ***p > 0.001; ****p < 0.0001 compared to Cont cells. *, **, **** in red significance obtained using an unpaired T-test (assuming Gaussian distribution) between Cont (untreated) and T2-KD (untreated) only

Next, we evaluated the response of T2-KD FT282, JOSH2, OVCAR4 cells and their corresponding controls to cisplatin and paclitaxel-based chemotherapy, standard drugs used for the cure of ovarian cancer patients. All three cell lines showed enhanced sensitivity to paclitaxel and cisplatin in response to T2-KD as shown by the reductions in IC_50_ values (50% cell killing) (7A). The T2-FT282 cell line was more sensitive to both chemotherapeutic agents than the cancer cell lines. The IC_50_ value in the T2-KD FT282 cell line was reduced 800–1000 fold by both paclitaxel and cisplatin treatments (Fig. 7a). However, in the OVCAR4 cell line there was a 5–6 fold decrease in the IC_50_ values in chemotherapy-treated T2-KD cells compared to the corresponding control cells (Fig. 7a). T2-KD JHOS2 cell line showed 3–5-fold lower IC_50_ values in response to chemotherapy treatment compared to its matched control (Fig. 7a).

### Chemotherapy treatments regulated MT1-MMP and MMP-2 in a TIMP 2 dependent manner

To determine if chemotherapy treatments have any effect on the expression of TIMP-2, MT1-MMP and MMP-2, mRNA and protein levels were evaluated in the OVCAR4 cells before and after chemotherapy treatments and TIMP-2 knock down. Treatment of OVCAR4 cells with individual IC_50_ doses of paclitaxel or cisplatin significantly enhanced TIMP-2 mRNA and protein levels compared to untreated Cont cells (Fig. 7b). However, this response to paclitaxel and cisplatin was not observed in T2-KD cells as TIMP2 was expressed at only basal levels following TIMP2 knock down (Fig. 7b).

Consistent with our earlier experiments (Fig. 4a), the basal protein expression of MT1-MMP was higher and MMP-2 was lower in T2-KD compared to Cont OVCAR4 cells in the absence of paclitaxel or cisplatin (ie untreated control – column 1 compared to column 4, Fig. 7c). As in the case of TIMP-2, both paclitaxel or cisplatin treatment substantially increased MT1-MMP and MMP-2 mRNA and protein levels in Cont cells (Fig. 7c, columns 2 and 3 compared to column 1 in each graph). In contrast, while MT1-MMP and MMP-2 mRNA still increased in T2-KD cells in response to chemotherapy, this effect was substantially blunted in T2-KD cells compared to Cont cells (Fig. 7c, columns 5 and 6 compared to 4 in each graph). Similarly, while MMP-2 protein increased in T2-KD cells in response to cisplatin and paclitaxel, this response was blunted by TIMP-2 knock down. Interestingly, this TIMP-2 knock down dependent blunting effect appeared more pronounced in paclitaxel treatment group than in the cisplatin treatment (Fig. 7c). While the MT1-MMP protein levels appeared reduced in T2-KD cells compared to the cisplatin or paclitaxel treated Cont cells, there was no difference between the untreated T2-TD cells compared to those treated with paclitaxel or cisplatin (Fig. 7c).

### Chemotherapy treatments enhanced chemoresistance and CSC markers in a TIMP2 dependent manner

**Fig. 8:**
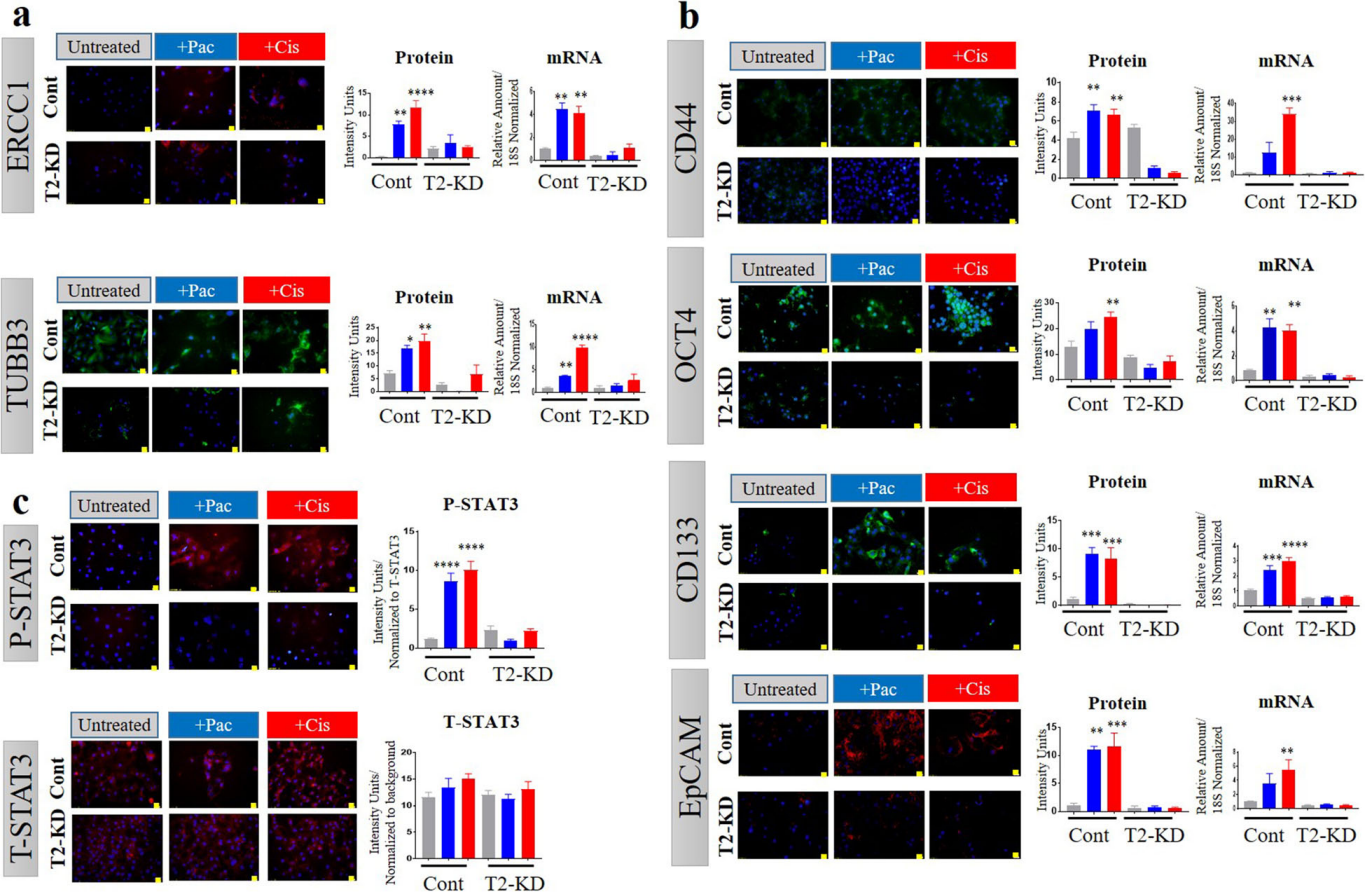
Effects of paclitaxel (pac) and cisplatin (cis) on the expression of chemoresistant genes (ERCC1 and TUBB3), and cancer stem cell markers (CD44, Oct4A, CD133 and EpCAM) in OVCAR4 Cont and T2-KD cells. The mRNA and protein expression of **a** ERCC1 (red) and TUBB3 (green), **b** CD44 (green), Oct4A (green), CD133 (green) and EpCAM (red), and **c** P-STAT3 (red) and T-STAT3 (red) in OVCAR4 Cont and T2-KD OVCAR4 cells treated with their respective IC_50_ doses of cis and pac were determined by immunofluorescence and relative qRT-PCR (untreated cells grey, cis treated red and pac treated, blue). Each experiment was repeated three times and was performed in triplicate. For immunofluorescence images, magnification was (20X); scale bar (in yellow) 20 μM. Significance was determined by one-way ANOVA *p > 0.05; **p > 0.01; ***p > 0.001; ****p < 0.0001 in Cont vs T2-KD cells

In order to explore further the mechanisms by which knock down of TIMP-2 increased chemosensitivity in OVCAR4 cells, we evaluated the expression of two chemoresistance markers, DNA excision repair complex protein 1 (ERCC1) and tubulin β type III (TUBB3), and cancer stem cell (CSC) markers [Pou5f1 (OCT4), CD44, CD133 and EpCAM)] at the mRNA and protein levels. The expression of both chemoresistance and all CSC markers were significantly enhanced at the mRNA and protein levels in Cont OVCAR4 cells in response to either chemotherapy treatment (Fig. 8a and b). However, the enhancement level of chemoresistance and CSC markers was substantially blunted in T2-KD OVCAR4 cells compared to Cont OVCAR4 cells, strongly indicating that the upregulation of these chemoresitance and CSC markers in response to either chemotherapy agent is TIMP-2 dependent.

### Chemotherapy treatment enhanced STAT3 phosphorylation in a TIMP2 dependent manner

We previously reported that phosphorylation of STAT3 (P-STAT3) occurs in response to chemotherapy treatments in ovarian cancer cell lines [18, 19, 31]. Consistent with our previous findings, OVCAR4 cells exhibited significantly enhanced STAT3 phosphorylation in response to either paclitaxel or cisplatin treatment (Fig. 8c). However, this response was abolished when TIMP-2 was reduced in the T2-KD OVCAR4 cells (Fig. 8c). The level of total STAT3 (T-STAT3) remained unchanged in response to all treatments.

### Chemotherapy dependent upregulation of chemoresistance and CSC markers requires STAT3 signalling and is correlated with a loss of TIMP-2 expression

**Fig. 9:**
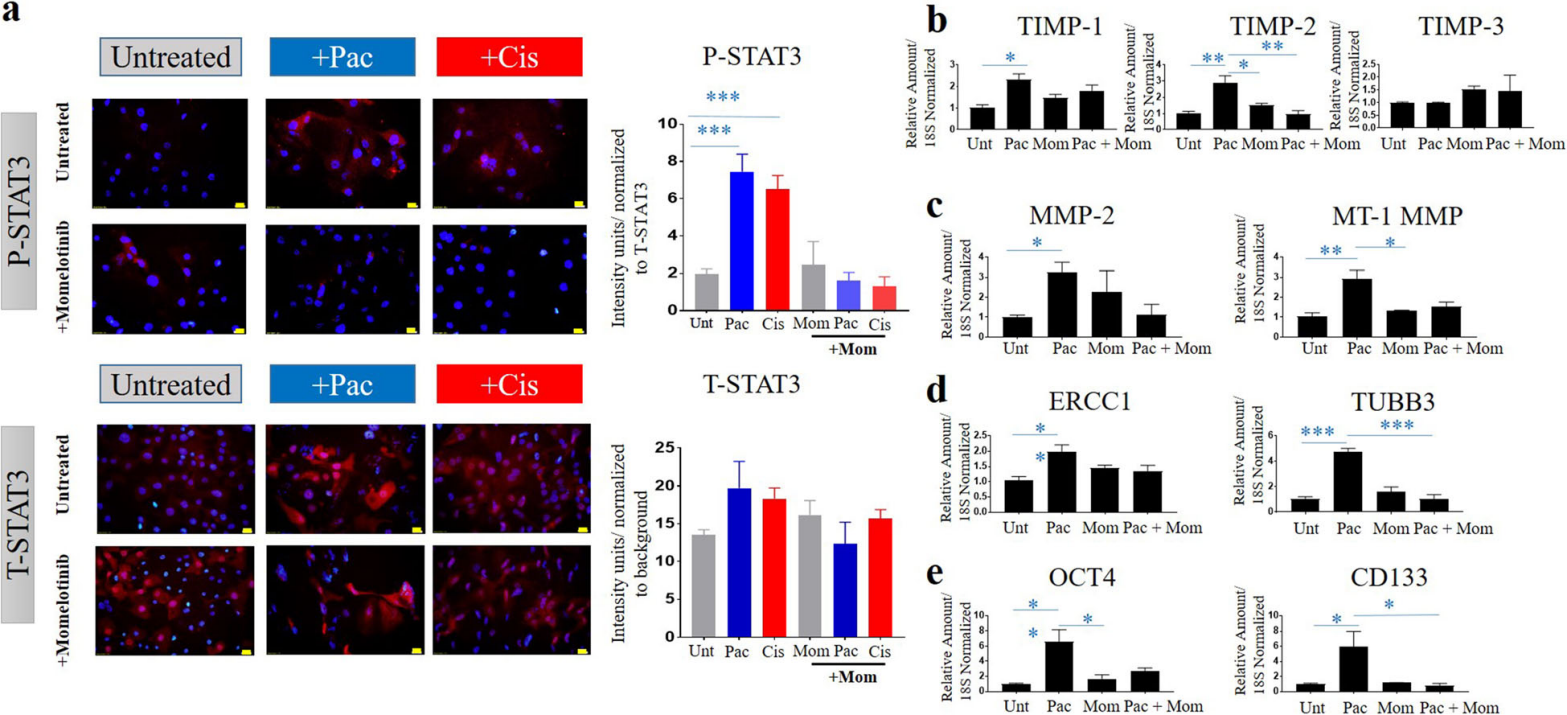
Effect of Momelotinib (Mom) on the expression of phospho and total STAT3, TIMP-1, 2 and 3, chemoresistant genes (ERCC1 and TUBB3), cancer stem cell markers (Oct4A, CD133) in OVCAR4 P cells treated with paclitaxel (pac) or cisplatin (cis). Effect of Momelotinib (1 μM) on **a** P-STAT3 (red) and T-STAT3 (red) expression after treatment with paclitaxel or cisplatin; **b** mRNA expression of TIMPs 1–3, **c** MMP-2 and MT1-MMP, **d** ERCC1, TUBB3 and **e** OCT4 and CD133 in OVCAR4 cells after treatment with paclitaxel only. P-STAT3 expression was evaluated by immunofluorescence. mRNA expression of TIMPs (1–3), MMP-2, MT1-MMP, ERCC1, TUBB3, OCT4A, CD133 was determined by qRT-PCR as described in Methods. Each experiment was repeated three times and was performed in triplicate. For immunofluorescence images, magnification was (20X); scale bar (in yellow) 20 μM. Significance was determined by one-way ANOVA *p > 0.05; **p > 0.01; ***p > 0.001 in OVCAR4 cells +/− treatment with paclitaxel or a combination of paclitaxel and Momelotinib

We next used a JAK2/STAT3 specific inhibitor, Momelotinib, to determine whether chemotherapy dependent upregulation of chemoresistance and CSC and markers depended on STAT3 signalling in OVCAR4 P cells. Momelotinib specifically inhibited cisplatin and paclitaxel-induced STAT3 phosphorylation in OVCAR4 cells (Fig. 9a). Moreover, Momelotinib also reduced paclitaxel-induced expression of TIMP-2 (Fig. 9b), MT1-MMP, MMP-2 (Fig. 9c), the expression of chemoresistance makers ERCC1 and TUBB3 (Fig. 9d) and the CSC markers OCT4 and CD133 (Fig. 9e) strongly indicating that chemotherapeutic induction of these markers requires STAT3 signalling.

In order to explore if paclitaxel-induced P-STAT3 also regulates the expression of TIMP-1 and TIMP-3, mRNA expression of TIMP-1 and TIMP-3 was evaluated on OVCAR4 paclitaxel and Momelotinib treated cells. We demonstrate that besides TIMP-2, paclitaxel significantly enhanced the mRNA expression of TIMP-1 but had no effect on the expression of TIMP-3 (Fig. 9b). However, while the inhibitory effect of Momelotinib was observed on paclitaxel-induced TIMP-2 expression, the expression of TIMP-1 and TIMP-3 remained unaffected (Fig. 9b). These results suggest that chemotherapy induced enhancement in P-STAT3 activation is associated with TIMP-2 and is independent of TIMP-1 and TIMP-3 expression.

## Discussion

There has been a substantial progress in the last few years in understanding the biological role of MMPs and their endogenous tissue inhibitors (TIMPs) in tumour progression [41, 6, 42]. It is now evident that TIMP-2, defined as a tissue inhibitor of MMPs, plays an important regulatory role in tumour biology through both MMP-inhibitory and MMP-independent mechanisms [7, 43, 44]. In this study, we demonstrate that TIMP-2 expression in high-grade serous ovarian tumours is significantly higher than in benign tumours of the same origin. We also demonstrate for the first time that suppression of TIMP-2 expression in Fallopian tube secretory epithelial cells and two ovarian cancer cell lines by an in vitro transient knock down (siRNA) modulates ovarian cell proliferation, invasion and sensitivity to chemotherapy.

The expression of TIMP-2 coincided mostly with the expression of CA125 in high-grade serous tumours, indicating that TIMP-2 is mainly expressed by epithelial tumour cells. However, some diffuse stromal staining of TIMP-2 was also evident in some malignant tumours. In benign tumours, weak expression of TIMP-2 was confined to the ovarian surface epithelium. Previous reports on the expression of TIMP-2 in ovarian tumours and its prognostic impact on the clinical outcome in patients have been ambiguous [45–48]. These indefinite findings regarding the expression of TIMP-2 in ovarian tumours may be due to different affinities of the antibodies used and differences in the experimental methods used for analysis of the studied protein. Alternatively, it could be due to differences in the pathology of high-grade serous tumours as distinct histopathological and genetically different sub-types of high-grade serous tumours have been described [49, 50].

Since our study demonstrated higher expression of TIMP-2 in high-grade serous ovarian tumours, we used normal secretory Fallopian tube and cancer cell lines that expressed relatively more TIMP-2 than TIMP-1 or TIMP-3, to investigate in vitro the cellular functions of TIMP-2 by transient knockdown. We transiently suppressed the TIMP-2 expression by siRNA in a Fallopian tube secretory epithelial cell line, FT282, and two ovarian cancer cell lines, JHOS2 and OVCAR4. Suppression of TIMP-2 in vitro in the three cell lines was associated with an increase in MT1-MMP expression but inhibition of MMP-2 expression and enzyme activation. Enhanced expression of MT1-MMP and the loss of expression and activation of MMP-2 in response to suppression of TIMP-2 may result due to unavailability of TIMP-2 to bind to MT1-MMP to initiate the activation MMP-2.

We also report for the first time that the suppression of TIMP-2 in ovarian cancer cell lines is associated with a loss of E-Cad mRNA expression with a corresponding increase in the mRNA expression of N-Cad, VIM and a decrease in the mRNA expression of COL12A1. These results are consistent with the study in a non-small cell lung cancer model which showed that overexpression of TIMP-2 upregulated E-Cad expression in in vitro and in vivo models, contributing to the maintenance of cell-cell adhesion and inhibition of tumour growth [44, 51]. Another study using gastric carcinoma cells has shown an inverse relationship between MT1-MMP expression and E-Cad expression, where knock down of MT1-MMP resulted in increased E-Cad expression which was related to inhibition of proliferation and invasion of gastric cancer cells [37]. The loss of E-Cad expression in response to suppression of TIMP-2 expression in ovarian cancer cells in our study did not lead to a change in the expression of classical EMT transcription factors downstream of E-Cad such as *SLUG*, *SNAIL* and *TWIST*. However, a decrease in *COL12A1* (alpha chain of type XII collagen, associated with type I collagen) mRNA expression in response to TIMP-2 suppression may be correlated with increased expression of MT1-MMP, a potent protease involved with the degradation of ECM-related fibrillar collagen implicated in tissue remodelling [39]. In this context, it should be mentioned that the mesothelial layer of the peritoneum is rich in interstitial collagen that provides structural support for optimum tissue assembly and hinders foreign implantation [52]. MT1-MMP is also essential for the release of ovarian cancer cells (either as sheets of cells or single cells) from primary tumours, which later accumulate as multicellular aggregates in the peritoneum prior to attachment on the mesothelial lining of the peritoneum [53]. MT1-MMP also promotes migration, cell-matrix detachment, ECM invasion, angiogenesis, formation of multicellular aggregates and growth in three-dimensional collagen matrices in ovarian cancer cells [54]. In addition, active MT1-MMP facilitates the shedding of ectodomain of MUC16/CA125 in ovarian cancer which restrains adhesion and invasion of cancer cells to the peritoneum [55]. These MT1-MMP-mediated functions may explain the enhanced invasion observed in the ovarian cancer cell lines with decreased TIMP-2 expression after siRNA treatment. Enhanced proliferation in response to TIMP-2 suppression may result due to enhancement in the expression of cell cycle analogues CDC25B and CDC25C, which may drive cells through G2 and M phases [56]. However, in the FT282 cell line, knockdown of TIMP-2 may only enhance the M2 phase of the cell cycle through CDC25B. This indicates that TIMP-2 may mediate proliferation in FT282 and ovarian cancer cell lines through different cell cycle mediated mechanisms.

Enhanced proliferation and invasion by knockdown of TIMP-2 in ovarian cancer cell lines is consistent with previous studies that have shown that overexpression of TIMP-2 reduced invasion and proliferation in cells. In melanoma B16F10 cell line TIMP-2 overexpression reduced invasion and angiogenic abilities of these cells [57]. Overexpression of TIMP-2 in rat smooth muscle cells produced a dose-dependent reduction in proliferation [58]. Consistent with that study, silencing miR939 produced an overexpression of endogenous TIMP-2 with consequent significant loss of proliferation of non-small cell lung cancer cell line (NSCLC) [59].

We have previously reported that both paclitaxel and cisplatin, standard chemotherapies used for the treatment of ovarian cancer patients, promotes an increase in the expression of the chemoresistant markers ERCC1 and TUBB3 and the CSC markers CD44, CD133, OCT4A and EpCAM in ovarian cancer cells, [16, 18, 19, 60]. In this study, we report similar findings in OVCAR4 cells. The increase in chemoresistance and CSC marker expression coincided with the upregulation of TIMP-2 expression and overlapped with the activation of STAT3 pathway in Cont OVCAR4 cells. However, T2-KD OVCAR4 cells, which had reduced TIMP-2 expression, did not exhibit activation of STAT3 or an increase in the expression of chemoresistance and CSC markers in response to chemotherapy treatments. These results may suggest that TIMP-2 and STAT3 activation are intrinsically associated with chemotherapy resistance in ovarian cancer. It can be speculated that the extracellular microenvironment initiated by the cytotoxic damage of the cancer cells may be associated with the activation of STAT3. In this context, the synthesis and secretion of cytokines like interleukin-6 (IL-6), a potent STAT3 activator, have been reported in response to cytotoxic damage in cancer cells [61]. In addition, the concentration of IL-6 increases after platinum treatment of ovarian tumours, and IL-6 secreted by stromal fibroblasts activates STAT3 and enriches the numbers of ALDH^+^ CSCs in residual tumours [38]. To date, no study has provided a drect link between TIMP-2, STAT3 and chemoresistance. However, a recent paper has demonstrated TIMP-1 mediated chemoresistance in a non-small cell lung carcinoma model via induction of IL-6 secretion [62]. In addition, in vitro TIMP-1 production in primary mouse hepatocytes was enhanced by IL-6 treatment, but it was less in STAT3-deficient hepatocytes [63].

Accumulating data suggests that the chemotherapy treated tumours facilitate the selection of therapy-resistant CSCs through elimination of sensitive cells, which makes the residual recurrent tumour aggressive and resistant to therapy [16, 64]. In addition, chemotherapy labile apoptotic/necrotic cells release intracellular metabolites and soluble cytokines/chemokines and growth factors which therapy-resistant cancer cells may require for the re-growth and establishment of recurrent tumours [4, 65, 66]. Furthermore, CSCs may escape host immune surveillance by upregulating checkpoint regulators [67]. The embryonic stem cell marker Nanog interacts with the cancer stem cell marker CD44 to activate the STAT3 pathway in ovarian cancer cells [68]. An enhanced expression of STAT3 has been reported in recurrent ovarian tumours extracted from metastatic ovarian lesions and ascites-derived tumours, compared to primary tumours and chemonaive ascites-derived tumours [69, 70]. The genomic and proteomic signatures of recurrent ovarian tumours have been associated with CSCs [21, 69]. Analyses of clinical samples have shown that the expression of CSC markers (CD44, CD133 and ALDH1A) is low in primary tumours but is enhanced in tumours immediately after chemotherapy treatment but reduces back to their original levels at recurrence, suggesting that the initial expression of CSCs markers identifies chemoresistant cells [71]. Our recent study has shown that daily oral treatment with Momelotinib (a potent JAK2/STAT3 inhibitor) as a maintenance treatment in conjunction with chemotherapy suppresses STAT3 activation, the CSC traits, and extends the disease-free period by deterring peritoneal spread in a mouse model of ovarian cancer [31]. Our current study also highlights the important role of the activated STAT3 pathway in CSC-mediated chemoresistance whereby we show a lack of an active STAT3-associated CSC pathway in T2-KD cells.

We report that a STAT3 inhibitor, Momelotinib, inhibits P-STAT3 activation in parental OVCAR4 cells resulting in loss of paclitaxel-induced TIMP-2 upregulation and concomitant loss in the enhancement of the expression of chemoresistance and CSC markers. Although paclitaxel enhanced TIMP-1 expression on parental OVCAR4 cells, Momelotinib had no effect on its expression, strongly suggesting that TIMP-2 mediated chemotherapy-induced STAT3 activation is essential for chemoresistant and CSC phenotypes in ovarian cancer.

TIMPs are secreted by normal and tumour cells and most likely have paracrine effects on the cells in the cellular microenvironment. It is possible that high expression of TIMP-2 in tumours is a pre-requisite for constitutive activation of STAT3 in many cancers or vice versa*,* constitutive activation of STAT3 may sustain enhanced TIMP-2 expression in certain cancers. In that context, we have demonstrated persistent activation of STAT3 in advanced-stage ovarian cancer [72]. Nuclear existence of activated (phosphorylated) STAT3 has been observed in 70% of advanced-stage ovarian cancer and that has been associated with decreased survival [25]. Whether this persistent STAT3 activation is responsible for enhanced expression of TIMP-2 in ovarian tumours or enhanced expression of TIMP-2 drives constitutive STAT3 activation remains to be determined. In the chemotherapy treatment scenario, it is possible that the chemotherapy-induced acute secretory process, which causes the release of soluble factors (including TIMP-2), may control the activation of STAT3 in ovarian cancer cells. In T2-KD OVCAR4 cells, this aspect of the secretory process may have been compromised resulting in the inability of the cells to activate the STAT3 pathway resulting in the loss of a chemoresistant population. This suggested role of TIMP-2 requires further investigation.

## Conclusion

**Fig. 10:**
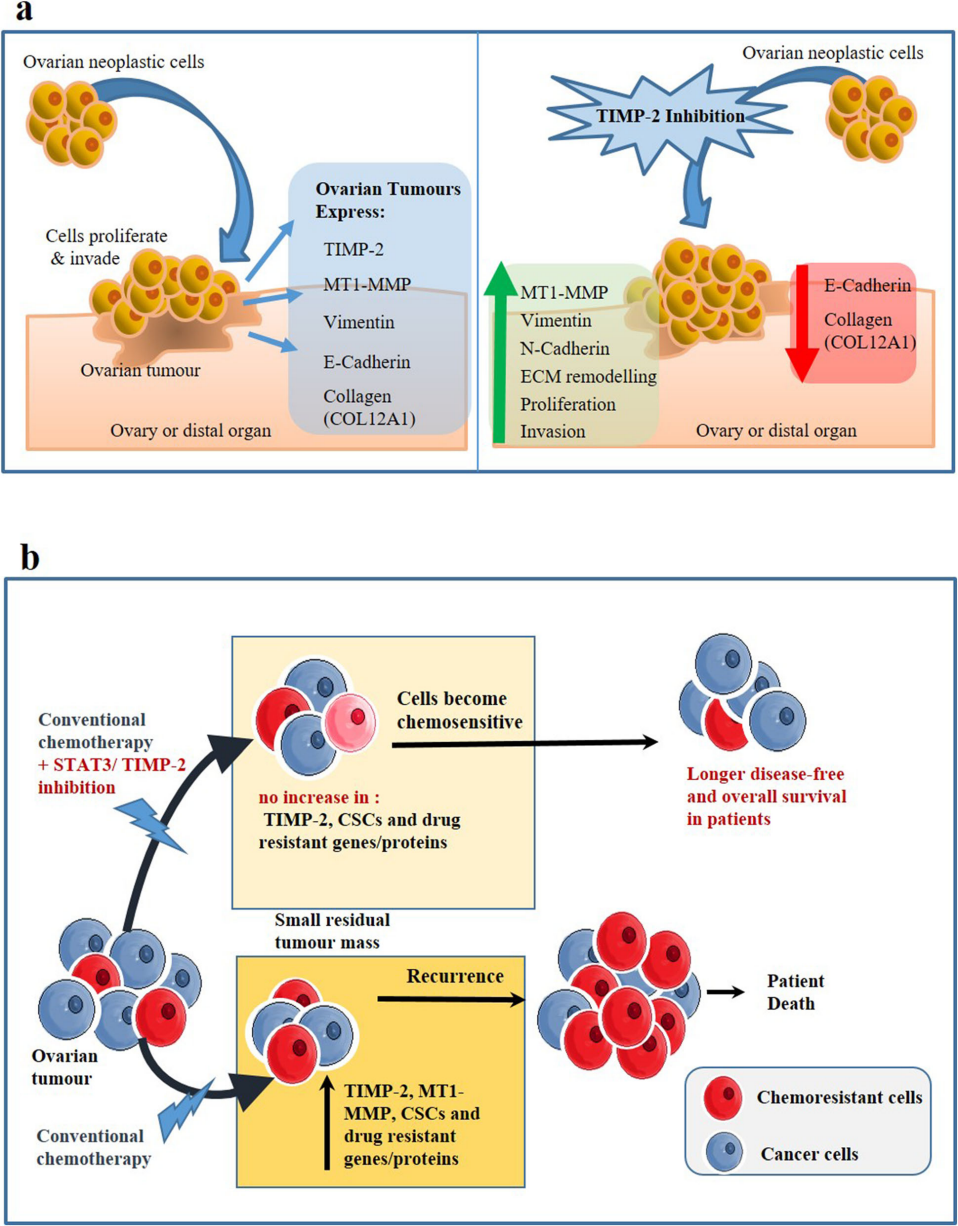
Proposed model of TIMP-2 inhibition in ovarian cancer cells. **a** Effect of TIMP-2 inhibition on proliferation and invasion: High-grade ovarian tumours overexpress TIMP-2. However, TIMP-2 inhibition increases proliferation and invasion of ovarian cancer cells. It is postulated to occur through enhancement in the expression of MT1-MMP, which facilitates ECM remodelling through downregulation of E-Cadherin expression, degradation of COL12A1 and upregulation of N-Cadherin and Vimentin. **b** Effect of TIMP-2 inhibition on chemoresistance: Chemotherapy treatment enhances TIMP-2 enriched CSC-like cells. We hypothesise that under the current conventional treatment protocol, which consists of platinum and taxane-based drugs most patients treated with chemotherapy undergo consecutive recurrences due to development of chemoresistant tumours enriched in TIMP-2 and CSCs. However, if the patients are treated with conventional chemotherapy in combination with STAT3 inhibitors or therapies that reduce TIMP-2 expression, this can eradicate CSCs during the first line of treatment, and/or subsequent lines of treatments. This consequently would lead to a decreased tumour burden with increased disease free survival periods and better treatment outcomes in ovarian cancer patients

The data from this study indicates opposing roles of TIMP-2 in ovarian tumourigenesis. In the first instance, we show that knock down of TIMP-2 in ovarian cancer cells results in enhanced proliferation and invasion, processes essential for the progression of cancer. However, we also demonstrate that knock down of TIMP-2 enhances the chemosensitivity of ovarian cancer cells, a process that potentially contradicts processes involved with tumourigenesis. We propose that the growth and invasive functions of TIMP-2 in ovarian tumour biology in vitro is MMP-dependent and are regulated by MT1-MMP, while the chemosensitivity aspect may be dependent on chemotherapy-induced activation of the STAT3 pathway. However, as both TIMP-2 and STAT3 are overexpressed in ovarian tumours, activated STAT3 and high TIMP-2 expression may play a dual role in maintaining in vivo ovarian tumourigenesis. Targeting the TIMP-2/STAT3 axis may provide a novel strategy to enhance the efficacy of current chemotherapy regimens, resulting in a much-needed better clinical outcome for this lethal disease. Based on our results, a proposed model of TIMP-2 regulating ovarian cancer proliferation, invasion and chemotherapy-induced chemoresistance is described in Fig. 10.

## Supplementary information

**Additional file 1: Figure S1.** Single fluorophore image corresponding to the immunofluorescence expression of TIMP-2, MT1-MMP and MMP-2 in FT282, OVCAR4 and JOSH2 cell lines shown in Figure 2b. The images were obtained as described in Figure 2b. Magnification 20X; scale bar (in yellow) 20 μM.

**Additional file 2: Figure S2.** Single fluorophore image corresponding to the expression of TIMP-2, in P, Cont and T2-KD cells derived from FT282, OVCAR4 and JOSH2 cell lines as represented in Figure 3a. The images were obtained as described in Figure 3a. Magnification 20X; scale bar (in yellow) 20 μM.

**Additional file 3:** **Figure S3.** Reduction of TIMP-2 by siRNA. TIMP-2 was knocked down by predesigned three 27mer small interfering RNA (siRNA A, B, C) duplexes and a pooled siRNA (ABC) directed against TIMP-2 in FT282, JOSH-2 and OVCAR4 cell lines as described in the Methods. (A) mRNA expression of TIMP-2, (B-C) TIMP-1 and 3 was determined by qRT-PCR as described in Methods. Each experiment was repeated three times and was performed in triplicate. Significance was determined by one-way ANOVA *p > 0.05; **p > 0.01; ***p > 0.001; ****p < 0.0001.

**Additional file 4: Figure S4.** Effect of TIMP-2 knock down on the expression of SLUG, SNAIL and TWIST in FT282 and ovarian cancer cell lines. The mRNA expression of SLUG, SNAIL and TWIST in FT282, JHOS2 and OVCAR4 cell lines was evaluated by qRT-PCR. The experiment was repeated three times in triplicate. Error bars are presented as mean ± of SEM.

**Additional file 5: Figure S5:** Quantification of EdU staining in Cont, P and T2-KD FT282, JOSH2 and OVCAR4 cells. Cells were stained with EdU and PI as described in the Methods. Flow cytometer representation of percentage of EdU stained cells in S-phase of the cell cycle.

**Additional file 6: Table S1.** Microscope filters used for the Immunofluorescence study

## Data Availability

The data presented in the study are not publicly available as it is a part of a PhD dissertation still in progress. However, the data can be made available from the corresponding author on a reasonable request.

## Abbreviations

TIMP: Tissue inhibitor of metalloproteinases
HGSOC: High grade serous ovarian carcinoma
T2-KD: TIMP-2 knocked down cells
siRNA: Small interfering RNA
Cont: Scrambled vector transfected control cells
P: Parental cells treated with transfection reagent
Unt: Untreated cells
MMP: Matrix metalloproteinase
MT1-MMP: Membrane type 1 metalloproteinase
CSCs: Cancer stem cells
JAK2: Janus activated kinase
STAT3: Signal transducer and activator of transcription 3
ERCC1: Excision repair cross-complementation group 1
TUBB3: Tubulin beta 3 class III
EpCAM: Epithelial cell adhesion molecule
E-Cad: E-cadherin
N-cad: N-cadherin
VIM: Vimentin
EMT: Epithelial mesenchymal transition
CA125: Cancer antigen125
MTT: 3-(4,5-dimethylthiazol-2-yl)-2,5-diphenyltetrazolium bromide
EdU: 5-Ethynyl-2′-deoxyuridine
SDS-PAGE: Sodium dodecyl sulfate-polyacrylamide gel electrophoresis
DAPI: 4′,6-diamidino-2-phenylindole
qRT-PCR: Quantitative Reverse Transcription Polymerase Chain Reaction

## References

[1] Lheureux S, Braunstein M, Oza AM (2019). Epithelial ovarian cancer: evolution of management in the era of precision medicine. CA Cancer J Clin.

[2] Freimund AE, Beach JA, Christie EL, Bowtell DDL (2018). Mechanisms of drug resistance in high-grade serous ovarian Cancer. Hematol Oncol Clin North Am.

[3] Norouzi-Barough L, Sarookhani MR, Sharifi M, Moghbelinejad S, Jangjoo S, Salehi R (2018). Molecular mechanisms of drug resistance in ovarian cancer. J Cell Physiol.

[4] Ahmed N, Escalona R, Leung D, Chan E, Kannourakis G (2018). Tumour microenvironment and metabolic plasticity in cancer and cancer stem cells: perspectives on metabolic and immune regulatory signatures in chemoresistant ovarian cancer stem cells. Semin Cancer Biol.

[5] Page-McCaw A, Ewald AJ, Werb Z (2007). Matrix metalloproteinases and the regulation of tissue remodelling. Nat Rev Mol Cell Biol.

[6] Apte SS, Parks WC (2015). Metalloproteinases: a parade of functions in matrix biology and an outlook for the future. Matrix Biol.

[7] Chirco R, Liu XW, Jung KK, Kim HR (2006). Novel functions of TIMPs in cell signaling. Cancer Metastasis Rev.

[8] Escalona RM, Chan E, Kannourakis G, Findlay JK, Ahmed N. The Many Facets of Metzincins and Their Endogenous Inhibitors: Perspectives on Ovarian Cancer Progression. Int J Mol Sci. 2018;19(2):450. 10.3390/ijms19020450 PMC5855672 29393911

[9] Toth M, Bernardo MM, Gervasi DC, Soloway PD, Wang Z, Bigg HF, Overall CM, DeClerck YA, Tschesche H, Cher ML (2000). Tissue inhibitor of metalloproteinase (TIMP)-2 acts synergistically with synthetic matrix metalloproteinase (MMP) inhibitors but not with TIMP-4 to enhance the (membrane type 1)-MMP-dependent activation of pro-MMP-2. J Biol Chem.

[10] Shen Q, Lee ES, Pitts RL, Wu MH, Yuan SY (2010). Tissue inhibitor of metalloproteinase-2 regulates matrix metalloproteinase-2-mediated endothelial barrier dysfunction and breast cancer cell transmigration through lung microvascular endothelial cells. Mol Cancer Res.

[11] Brew K, Nagase H (2010). The tissue inhibitors of metalloproteinases (TIMPs): an ancient family with structural and functional diversity. Biochim Biophys Acta.

[12] Hoegy SE, Oh HR, Corcoran ML, Stetler-Stevenson WG (2001). Tissue inhibitor of metalloproteinases-2 (TIMP-2) suppresses TKR-growth factor signaling independent of metalloproteinase inhibition. J Biol Chem.

[13] Fernandez CA, Roy R, Lee S, Yang J, Panigrahy D, Van Vliet KJ, Moses MA (2010). The anti-angiogenic peptide, loop 6, binds insulin-like growth factor-1 receptor. J Biol Chem.

[14] Seo DW, Li H, Guedez L, Wingfield PT, Diaz T, Salloum R, Wei BY, Stetler-Stevenson WG (2003). TIMP-2 mediated inhibition of angiogenesis: an MMP-independent mechanism. Cell.

[15] Sanchez-Pozo J, Baker-Williams AJ, Woodford MR, Bullard R, Wei B, Mollapour M, Stetler-Stevenson WG, Bratslavsky G, Bourboulia D (2018). Extracellular Phosphorylation of TIMP-2 by Secreted c-Src Tyrosine Kinase Controls MMP-2 Activity. iScience.

[16] Ahmed N, Abubaker K, Findlay JK (2014). Ovarian cancer stem cells: molecular concepts and relevance as therapeutic targets. Mol Aspects Med.

[17] Zhang S, Balch C, Chan MW, Lai HC, Matei D, Schilder JM, Yan PS, Huang TH, Nephew KP (2008). Identification and characterization of ovarian cancer-initiating cells from primary human tumors. Cancer Res.

[18] Abubaker K, Luwor RB, Escalona R, McNally O, Quinn MA, Thompson EW, Findlay JK, Ahmed N (2014). Targeted disruption of the JAK2/STAT3 pathway in combination with systemic Administration of Paclitaxel Inhibits the priming of ovarian Cancer stem cells leading to a reduced tumor burden. Front Oncol.

[19] Abubaker K, Luwor RB, Zhu H, McNally O, Quinn MA, Burns CJ, Thompson EW, Findlay JK, Ahmed N (2014). Inhibition of the JAK2/STAT3 pathway in ovarian cancer results in the loss of cancer stem cell-like characteristics and a reduced tumor burden. BMC Cancer.

[20] Jin W. Role of JAK/STAT3 Signaling in the Regulation of Metastasis, the Transition of Cancer Stem Cells, and Chemoresistance of Cancer by Epithelial-Mesenchymal Transition. Cells. 2020;9(1):217. 10.3390/cells9010217 PMC7017057 31952344

[21] Steg AD, Bevis KS, Katre AA, Ziebarth A, Dobbin ZC, Alvarez RD, Zhang K, Conner M, Landen CN (2012). Stem cell pathways contribute to clinical chemoresistance in ovarian cancer. Clin Cancer Res.

[22] Sherry MM, Reeves A, Wu JK, Cochran BH (2009). STAT3 is required for proliferation and maintenance of multipotency in glioblastoma stem cells. Stem Cells.

[23] Matthews JR, Sansom OJ, Clarke AR (2011). Absolute requirement for STAT3 function in small-intestine crypt stem cell survival. Cell Death Differ.

[24] Staniszewska AD, Pensa S, Caffarel MM, Anderson LH, Poli V, Watson CJ (2012). Stat3 is required to maintain the full differentiation potential of mammary stem cells and the proliferative potential of mammary luminal progenitors. PLoS One.

[25] Rosen DG, Mercado-Uribe I, Yang G, Bast RC, Amin HM, Lai R, Liu J (2006). The role of constitutively active signal transducer and activator of transcription 3 in ovarian tumorigenesis and prognosis. Cancer.

[26] Quintas-Cardama A, Verstovsek S (2013). Molecular pathways: Jak/STAT pathway: mutations, inhibitors, and resistance. Clin Cancer Res.

[27] Yamada K, Tachibana T, Hashimoto H, Suzuki K, Yanagida S, Endoh H, Kimura E, Yasuda M, Tanaka T, Ishikawa H (1999). Establishment and characterization of cell lines derived from serous adenocarcinoma (JHOS-2) and clear cell adenocarcinoma (JHOC-5, JHOC-6) of human ovary. Hum Cell.

[28] Matsumura N, Huang Z, Mori S, Baba T, Fujii S, Konishi I, Iversen ES, Berchuck A, Murphy SK (2011). Epigenetic suppression of the TGF-beta pathway revealed by transcriptome profiling in ovarian cancer. Genome Res.

[29] Pirker R, FitzGerald DJ, Hamilton TC, Ozols RF, Willingham MC, Pastan I (1985). Anti-transferrin receptor antibody linked to Pseudomonas exotoxin as a model immunotoxin in human ovarian carcinoma cell lines. Cancer Res.

[30] Karst AM, Drapkin R (2012). Primary culture and immortalization of human fallopian tube secretory epithelial cells. Nat Protoc.

[31] Chan E, Luwor R, Burns C, Kannourakis G, Findlay JK, Ahmed N (2018). Momelotinib decreased cancer stem cell associated tumor burden and prolonged disease-free remission period in a mouse model of human ovarian cancer. Oncotarget.

[32] Shield K, Riley C, Quinn MA, Rice GE, Ackland ML, Ahmed N (2007). Alpha2beta1 integrin affects metastatic potential of ovarian carcinoma spheroids by supporting disaggregation and proteolysis. J Carcinog.

[33] Murnane MJ, Shuja S, Del Re E, Cai J, Iacobuzio-Donahue C, Klepeis V (1997). Characterizing human colorectal carcinomas by proteolytic profile. In Vivo.

[34] Wakeling SI, Miles DC, Western PS (2013). Identifying disruptors of male germ cell development by small molecule screening in ex vivo gonad cultures. BMC Res Notes.

[35] Bilandzic M, Stenvers KL. Assessment of ovarian cancer spheroid attachment and invasion of mesothelial cells in real time. J Visualized Experiments. 2014;87(E51655):1-6. 10.3791/51655 PMC4199467 24893837

[36] Sarraj MA, Escalona RM, Western P, Findlay JK, Stenvers KL (2013). Effects of TGFbeta2 on wild-type and Tgfbr3 knockout mouse fetal testis. Biol Reprod.

[37] Li B, Lou G, Zhou J (2019). MT1MMP promotes the proliferation and invasion of gastric carcinoma cells via regulating vimentin and Ecadherin. Mol Med Rep.

[38] Sulaiman A, Yao ZM, Wang LS (2018). Re-evaluating the role of epithelial-mesenchymal-transition in cancer progression. J Biomed Res.

[39] Gifford V, Itoh Y (2019). MT1-MMP-dependent cell migration: proteolytic and non-proteolytic mechanisms. Biochem Soc Trans.

[40] Sur S, Agrawal DK (2016). Phosphatases and kinases regulating CDC25 activity in the cell cycle: clinical implications of CDC25 overexpression and potential treatment strategies. Mol Cell Biochem.

[41] Gialeli C, Theocharis AD, Karamanos NK (2011). Roles of matrix metalloproteinases in cancer progression and their pharmacological targeting. FEBS J.

[42] Tallant C, Marrero A, Gomis-Ruth FX (2010). Matrix metalloproteinases: fold and function of their catalytic domains. Biochim Biophys Acta.

[43] Stetler-Stevenson WG, Gavil NV (2014). Normalization of the tumor microenvironment: evidence for tissue inhibitor of metalloproteinase-2 as a cancer therapeutic. Connect Tissue Res.

[44] Bourboulia D, Jensen-Taubman S, Rittler MR, Han HY, Chatterjee T, Wei B, Stetler-Stevenson WG (2011). Endogenous angiogenesis inhibitor blocks tumor growth via direct and indirect effects on tumor microenvironment. Am J Pathol.

[45] Halon A, Nowak-Markwitz E, Donizy P, Matkowski R, Maciejczyk A, Gansukh T, Gyorffy B, Spaczynski M, Zabel M, Lage H (2012). Enhanced immunoreactivity of TIMP-2 in the stromal compartment of tumor as a marker of favorable prognosis in ovarian cancer patients. J Histochem Cytochem.

[46] Davidson B, Goldberg I, Gotlieb WH, Kopolovic J, Ben-Baruch G, Nesland JM, Reich R (2002). The prognostic value of metalloproteinases and angiogenic factors in ovarian carcinoma. Mol Cell Endocrinol.

[47] Okamoto T, Niu R, Yamada S (2003). Increased expression of tissue inhibitor of metalloproteinase-2 in clear cell carcinoma of the ovary. Mol Hum Reprod.

[48] Sakata K, Shigemasa K, Nagai N, Ohama K (2000). Expression of matrix metalloproteinases (MMP-2, MMP-9, MT1-MMP) and their inhibitors (TIMP-1, TIMP-2) in common epithelial tumors of the ovary. Int J Oncol.

[49] Ohsuga T, Yamaguchi K, Kido A, Murakami R, Abiko K, Hamanishi J, Kondoh E, Baba T, Konishi I, Matsumura N (2017). Distinct preoperative clinical features predict four histopathological subtypes of high-grade serous carcinoma of the ovary, fallopian tube, and peritoneum. BMC Cancer.

[50] Tothill RW, Tinker AV, George J, Brown R, Fox SB, Lade S, Johnson DS, Trivett MK, Etemadmoghadam D, Locandro B (2008). Novel molecular subtypes of serous and endometrioid ovarian cancer linked to clinical outcome. Clin Cancer Res.

[51] Bourboulia D, Han H, Jensen-Taubman S, Gavil N, Isaac B, Wei B, Neckers L, Stetler-Stevenson WG (2013). TIMP-2 modulates cancer cell transcriptional profile and enhances E-cadherin/beta-catenin complex expression in A549 lung cancer cells. Oncotarget.

[52] Moss NM, Barbolina MV, Liu Y, Sun L, Munshi HG, Stack MS (2009). Ovarian cancer cell detachment and multicellular aggregate formation are regulated by membrane type 1 matrix metalloproteinase: a potential role in I.p. metastatic dissemination. Cancer Res.

[53] Steinkamp MP, Winner KK, Davies S, Muller C, Zhang Y, Hoffman RM, Shirinifard A, Moses M, Jiang Y, Wilson BS (2013). Ovarian tumor attachment, invasion, and vascularization reflect unique microenvironments in the peritoneum: insights from xenograft and mathematical models. Front Oncol.

[54] Ellerbroek SM, Wu YI, Overall CM, Stack MS (2001). Functional interplay between type I collagen and cell surface matrix metalloproteinase activity. J Biol Chem.

[55] Bruney L, Conley KC, Moss NM, Liu Y, Stack MS (2014). Membrane-type I matrix metalloproteinase-dependent ectodomain shedding of mucin16/ CA-125 on ovarian cancer cells modulates adhesion and invasion of peritoneal mesothelium. Biol Chem.

[56] Yin Y, Dou X, Duan S, Zhang L, Xu Q, Li H, Li D (2016). Downregulation of cell division cycle 25 homolog C reduces the radiosensitivity and proliferation activity of esophageal squamous cell carcinoma. Gene.

[57] Valente P, Fassina G, Melchiori A, Masiello L, Cilli M, Vacca A, Onisto M, Santi L, Stetler-Stevenson WG, Albini A (1998). TIMP-2 over-expression reduces invasion and angiogenesis and protects B16F10 melanoma cells from apoptosis. Int J Cancer.

[58] Baker AH, Zaltsman AB, George SJ, Newby AC (1998). Divergent effects of tissue inhibitor of metalloproteinase-1, −2, or −3 overexpression on rat vascular smooth muscle cell invasion, proliferation, and death in vitro. TIMP-3 promotes apoptosis. J Clin Invest.

[59] Chen A, Liu S, Lu X, Wei L, Chen Y (2018). Inhibition of microRNA939 suppresses the development of human nonsmall cell lung cancer via the upregulation of tissue inhibitor of metalloproteinases 2. Mol Med Rep.

[60] Abubaker K, Latifi A, Luwor R, Nazaretian S, Zhu H, Quinn MA, Thompson EW, Findlay JK, Ahmed N (2013). Short-term single treatment of chemotherapy results in the enrichment of ovarian cancer stem cell-like cells leading to an increased tumor burden. Mol Cancer.

[61] Rodier F, Coppe JP, Patil CK, Hoeijmakers WA, Munoz DP, Raza SR, Freund A, Campeau E, Davalos AR, Campisi J (2009). Persistent DNA damage signalling triggers senescence-associated inflammatory cytokine secretion. Nat Cell Biol.

[62] Xiao W, Wang L, Howard J, Kolhe R, Rojiani AM, Rojiani MV. TIMP-1-Mediated Chemoresistance via Induction of IL-6 in NSCLC. Cancers (Basel). 2019;11(8):1184. 10.3390/cancers11081184 PMC6721590 31443242

[63] Wang H, Lafdil F, Wang L, Yin S, Feng D, Gao B (2011). Tissue inhibitor of metalloproteinase 1 (TIMP-1) deficiency exacerbates carbon tetrachloride-induced liver injury and fibrosis in mice: involvement of hepatocyte STAT3 in TIMP-1 production. Cell Biosci.

[64] Guddati AK (2012). Ovarian cancer stem cells: elusive targets for chemotherapy. Med Oncol.

[65] Levina V, Su Y, Nolen B, Liu X, Gordin Y, Lee M, Lokshin A, Gorelik E (2008). Chemotherapeutic drugs and human tumor cells cytokine network. Int J Cancer.

[66] Kareva I, Waxman DJ, Lakka Klement G (2015). Metronomic chemotherapy: an attractive alternative to maximum tolerated dose therapy that can activate anti-tumor immunity and minimize therapeutic resistance. Cancer Lett.

[67] Kareva I. A Combination of Immune Checkpoint Inhibition with Metronomic Chemotherapy as a Way of Targeting Therapy-Resistant Cancer Cells. Int J Mol Sci. 2017;18(10):2134. 10.3390/ijms18102134 PMC5666816 29027915

[68] Bourguignon LY, Peyrollier K, Xia W, Gilad E (2008). Hyaluronan-CD44 interaction activates stem cell marker Nanog, Stat-3-mediated MDR1 gene expression, and ankyrin-regulated multidrug efflux in breast and ovarian tumor cells. J Biol Chem.

[69] Ahmed N, Greening D, Samardzija C, Escalona RM, Chen M, Findlay JK, Kannourakis G (2016). Unique proteome signature of post-chemotherapy ovarian cancer ascites-derived tumor cells. Sci Rep.

[70] Duan Z, Foster R, Bell DA, Mahoney J, Wolak K, Vaidya A, Hampel C, Lee H, Seiden MV (2006). Signal transducers and activators of transcription 3 pathway activation in drug-resistant ovarian cancer. Clin Cancer Res.

[71] Okudela K, Woo T, Mitsui H, Tajiri M, Masuda M, Ohashi K (2012). Expression of the potential cancer stem cell markers, CD133, CD44, ALDH1, and beta-catenin, in primary lung adenocarcinoma--their prognostic significance. Pathol Int.

[72] Colomiere M, Ward AC, Riley C, Trenerry MK, Cameron-Smith D, Findlay J, Ackland L, Ahmed N (2009). Cross talk of signals between EGFR and IL-6R through JAK2/STAT3 mediate epithelial-mesenchymal transition in ovarian carcinomas. Br J Cancer.

