# Supplementary figures and images for: TIMP-2 regulates proliferation, invasion and STAT3-mediated cancer stem cell-dependent chemoresistance in ovarian cancer cells
Source: BMC Cancer. 2020 Oct 6;20:960. doi: 10.1186/s12885-020-07274-6 (PMC7542139; doi:10.1186/s12885-020-07274-6)

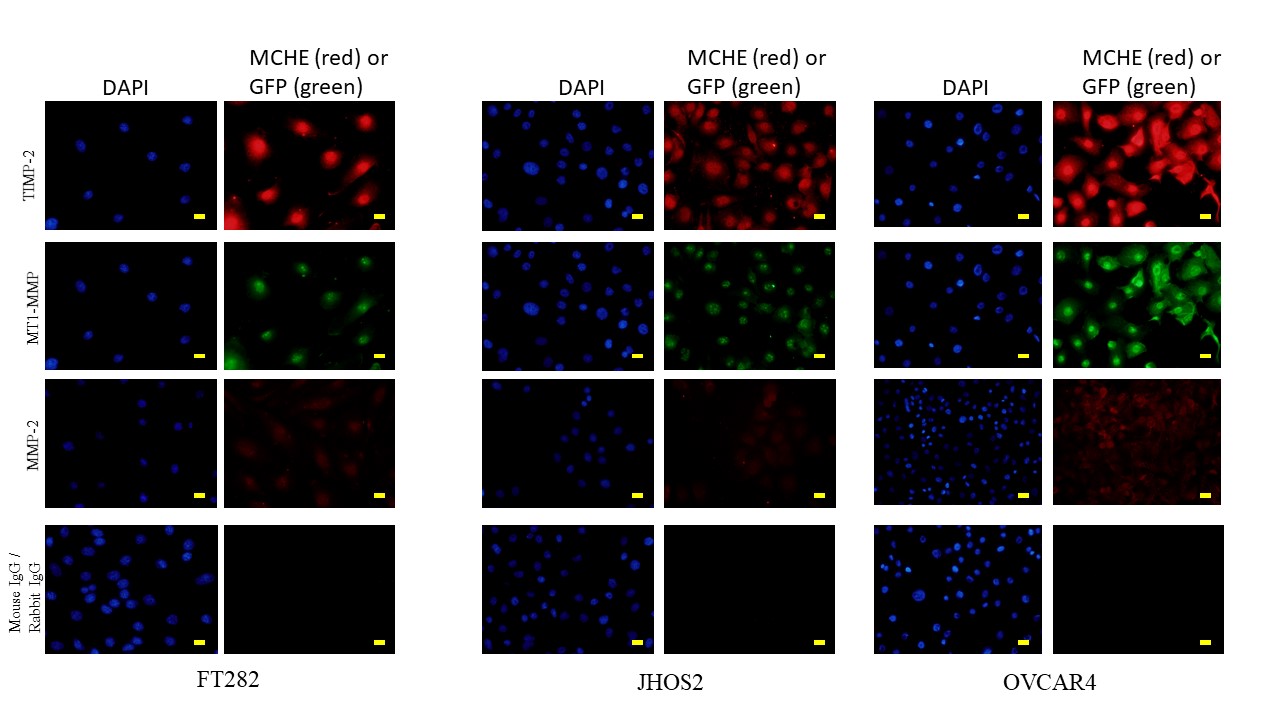

Supplement: Supplementary file 1 — Additional file 1: Figure S1. Single fluorophore image corresponding to the immunofluorescence expression of TIMP-2, MT1-MMP and MMP-2 in FT282, OVCAR4 and JOSH2 cell lines shown in Figure 2b. The images were obtained as described in Figure 2b. Magnification 20X; scale bar (in yellow) 20 μM. [file 12885_2020_7274_MOESM1_ESM.jpg]

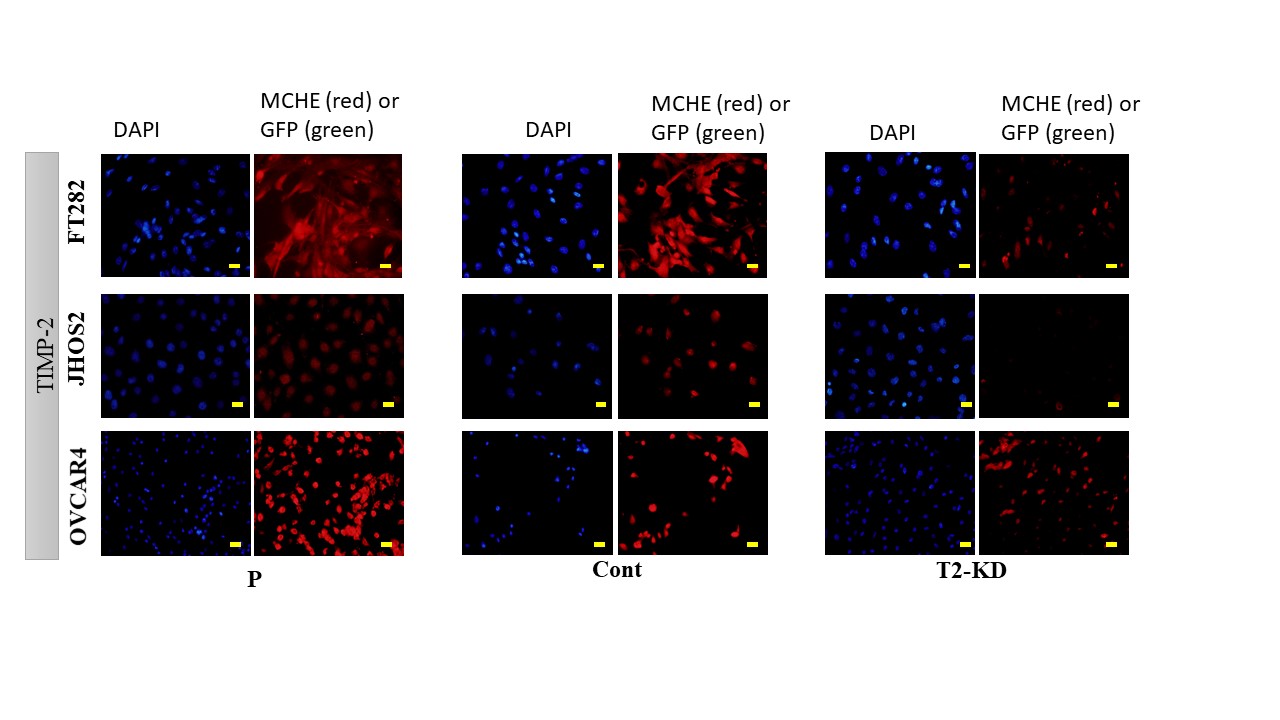

Supplement: Supplementary file 2 — Additional file 2: Figure S2. Single fluorophore image corresponding to the expression of TIMP-2, in P, Cont and T2-KD cells derived from FT282, OVCAR4 and JOSH2 cell lines as represented in Figure 3a. The images were obtained as described in Figure 3a. Magnification 20X; scale bar (in yellow) 20 μM. [file 12885_2020_7274_MOESM2_ESM.jpg]

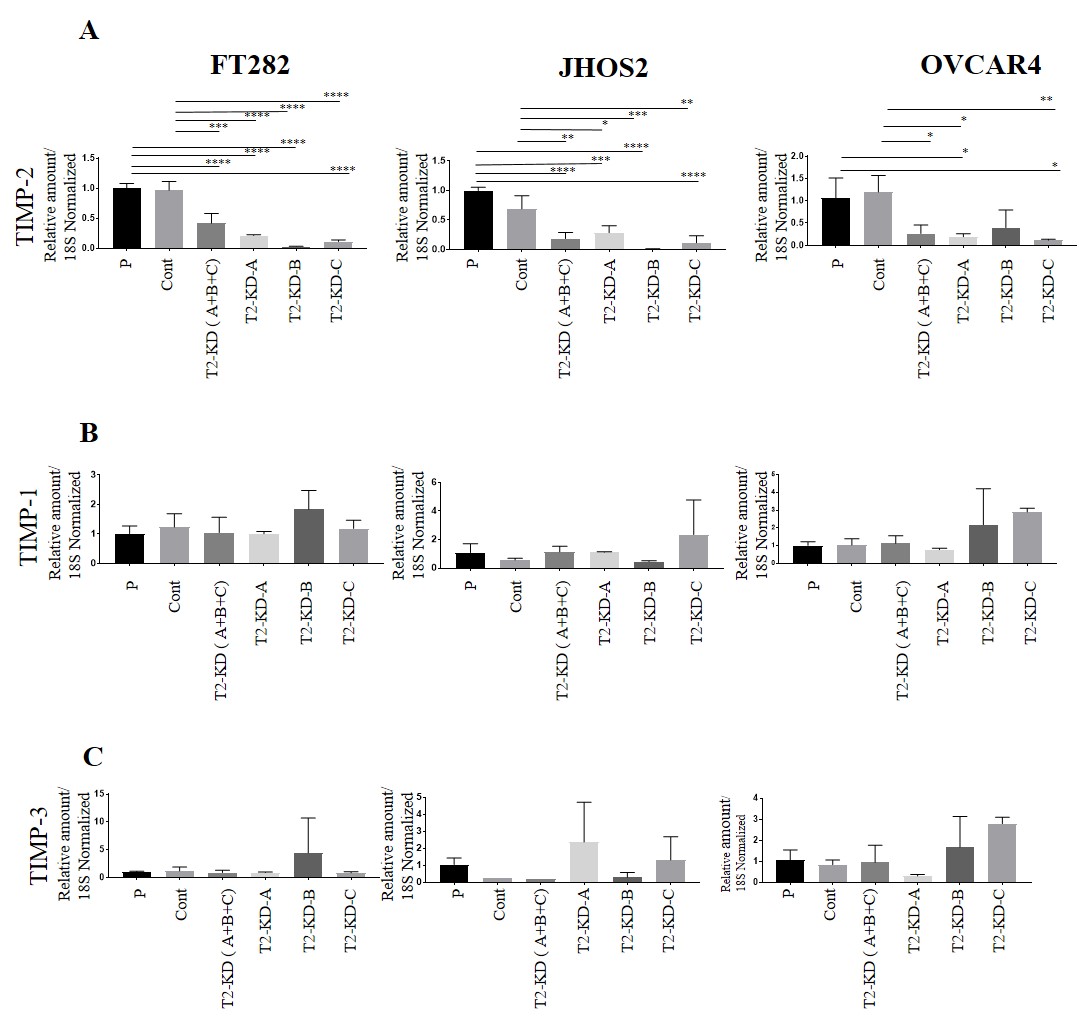

Supplement: Supplementary file 3 — Additional file 3: Figure S3. Reduction of TIMP-2 by siRNA. TIMP-2 was knocked down by predesigned three 27mer small interfering RNA (siRNA A, B, C) duplexes and a pooled siRNA (ABC) directed against TIMP-2 in FT282, JOSH-2 and OVCAR4 cell lines as described in the Methods. (A) mRNA expression of TIMP-2, (B-C) TIMP-1 and 3 was determined by qRT-PCR as described in Methods. Each experiment was repeated three times and was performed in triplicate. Significance was determined by one-way ANOVA *p > 0.05; **p > 0.01; ***p > 0.001; ****p < 0.0001. [file 12885_2020_7274_MOESM3_ESM.jpg]

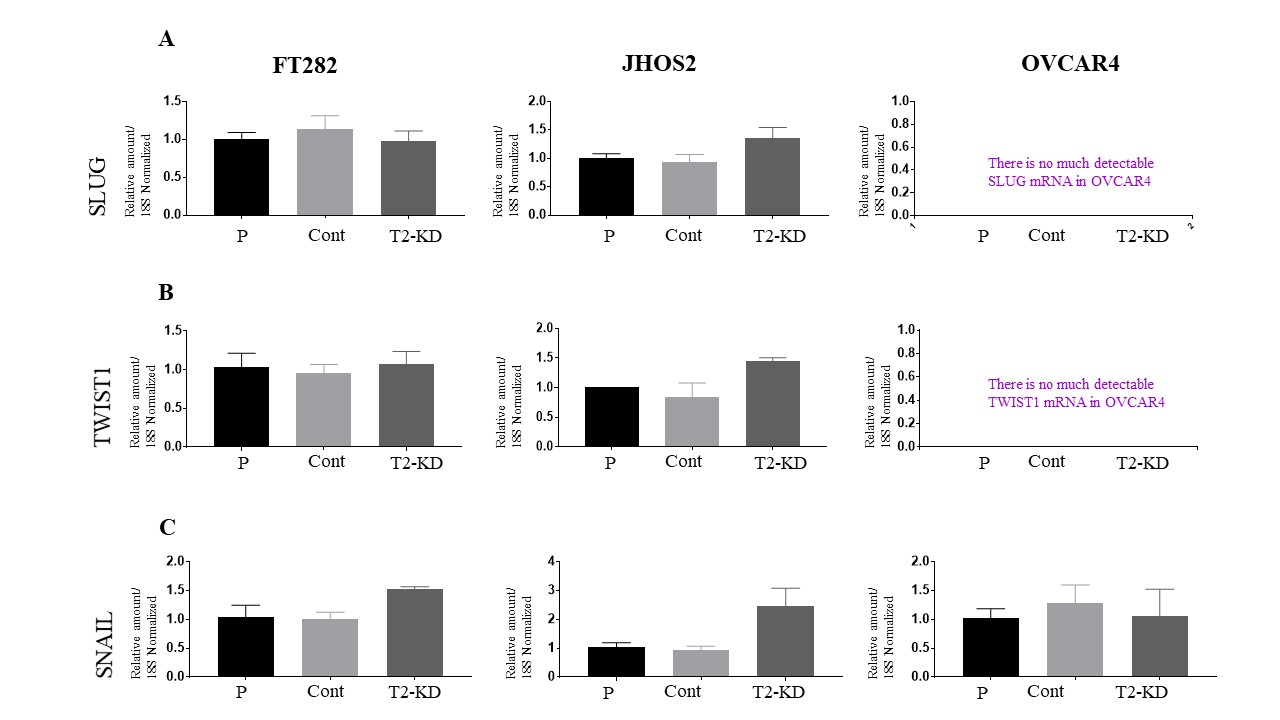

Supplement: Supplementary file 4 — Additional file 4: Figure S4. Effect of TIMP-2 knock down on the expression of SLUG, SNAIL and TWIST in FT282 and ovarian cancer cell lines. The mRNA expression of SLUG, SNAIL and TWIST in FT282, JHOS2 and OVCAR4 cell lines was evaluated by qRT-PCR. The experiment was repeated three times in triplicate. Error bars are presented as mean ± of SEM. [file 12885_2020_7274_MOESM4_ESM.jpg]

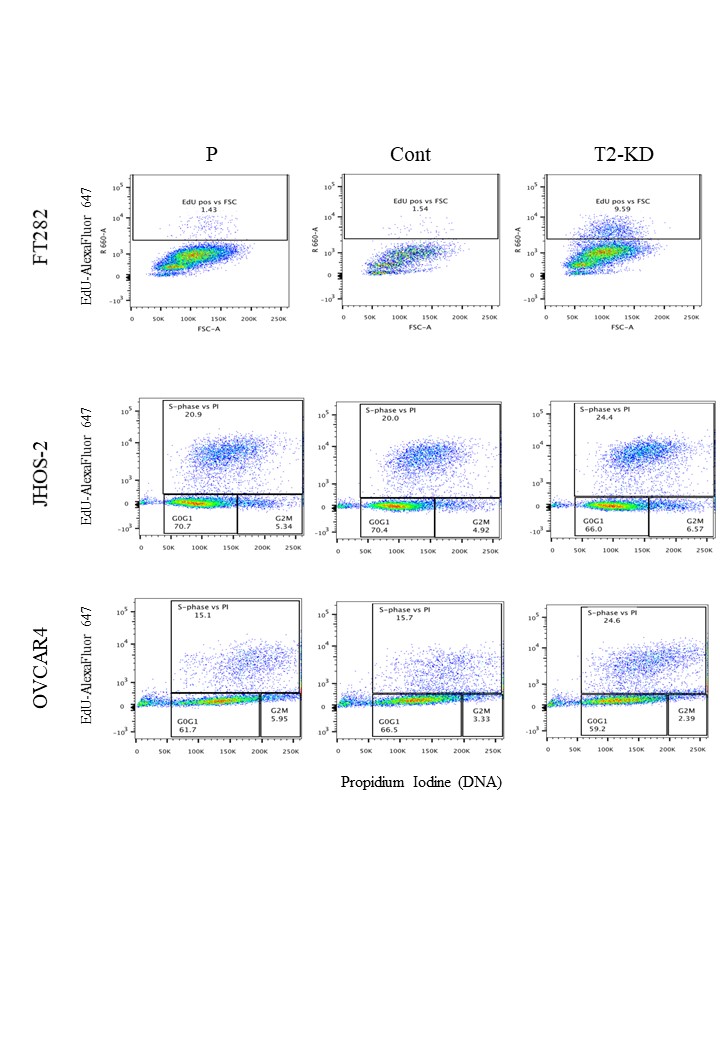

Supplement: Supplementary file 5 — Additional file 5: Figure S5: Quantification of EdU staining in Cont, P and T2-KD FT282, JOSH2 and OVCAR4 cells. Cells were stained with EdU and PI as described in the Methods. Flow cytometer representation of percentage of EdU stained cells in S-phase of the cell cycle. [file 12885_2020_7274_MOESM5_ESM.jpg]
