# Supplementary material for: TIMP-2 regulates proliferation, invasion and STAT3-mediated cancer stem cell-dependent chemoresistance in ovarian cancer cells
Source: BMC Cancer. 2020 Oct 6;20:960. doi: 10.1186/s12885-020-07274-6 (PMC7542139; doi:10.1186/s12885-020-07274-6)
Supplement: Supplementary file 6 — Additional file 6: Table S1. Microscope filters used for the Immunofluorescence study [file 12885_2020_7274_MOESM6_ESM.docx]

**Supplementary Table 1: Microscope filters used for the Immunofluorescence study**

| **Filter** | **Excitation**  **Wavelength (nm)** | **Emission**  **Wavelength (nm)** | **Fluorophore** |
| --- | --- | --- | --- |
| UW | 340-390 | 420 | DAPI |
| GFP | 460-480 | 495-540 | Alexa 488 |
| MCHE | 565-585 | 600-690 | Alexa 568, Alexa 594 |
